# Histone deacetylase (Rpd3) regulates *Drosophila* early brain development via regulation of Tailless

**DOI:** 10.1098/rsob.200029

**Published:** 2020-09-02

**Authors:** Paromita Das, Manika Pal Bhadra

**Affiliations:** 1 Applied Biology Division, CSIR-Indian Institute of Chemical Technology, Uppal Road, Hyderabad 500 007, India; 2 Academy of Scientific and Innovative Research (AcSIR) Training and Development Complex, CSIR Campus, CSIR Road, Taramani, Chennai 600 113, India

**Keywords:** gap genes, anteroposterior axis, loss-of-function, Tailless, brain development, *Drosophila*

## Abstract

Tailless is a committed transcriptional repressor and principal regulator of the brain and eye development in *Drosophila*. Rpd3, the histone deacetylase, is an established repressor that interacts with co-repressors like Sin3a, Prospero, Brakeless and Atrophin. This study aims at deciphering the role of *Rpd3* in embryonic segmentation and larval brain development in *Drosophila*. It delineates the mechanism of Tailless regulation by *Rpd3*, along with its interacting partners. There was a significant reduction in Tailless in *Rpd3* heteroallelic mutant embryos, substantiating that Rpd3 is indispensable for the normal Tailless expression. The expression of the primary readout, Tailless was correlative to the expression of the neural cell adhesion molecule homologue, Fascilin2 (Fas2). Rpd3 also aids in the proper development of the mushroom body. Both Tailless and Fas2 expression are reported to be antagonistic to the epidermal growth factor receptor (EGFR) expression. The decrease in Tailless and Fas2 expression highlights that EGFR is upregulated in the larval mutants, hindering brain development. This study outlines the axis comprising Rpd3, dEGFR, Tailless and Fas2, which interact to fine-tune the early segmentation and larval brain development. Therefore, Rpd3 along with Tailless has immense significance in early embryogenesis and development of the larval brain.

## 1. Introduction

Gap genes are the first subset of the zygotic genes in the *Drosophila* embryo that is activated under the influence of the maternal factors—Bicoid and Caudal. *Hunchback (hb)* and *Tailless (tll)* constitute the primary gap genes, solely dependent upon maternal genes while the secondary gap genes, *Krüppel (Kr)* and *giant (gt)*, perceive cues from the primary gap genes along with the maternal genes. Until the cellular blastoderm stage is reached, Tailless migrates from the nucleus to the cytoplasm. Subsequently, Tailless expresses only in the anterior embryonic region, forming the prospective brain: serving both as a repressor (as in the case of Kruppel) [1] and as an activator (in the case of Hunchback) [2].

The co-repressors, Heat shock factor (Hsf) and Tramtrack69 (Ttk69), together with GAGA factor (GAF) comprise the dual transcriptional switch that represses *tll* transcription by binding to the tor response element (tor-RE) at the *tll cis*-regulatory region. This repression of *tll* is relieved when the Torso (Tor) signalling pathway activates Mapk downstream that converts Hsf to an activator and degrades Ttk69 by phosphorylation [3]. *Tailless* represses genes from being activated by the epidermal growth factor receptor (EGFR) signalling and/or activates genes that block the reception or execution of such cues. Tailless ensures that the precursor cells achieve the optic lobe fate while a steep increase in EGFR shifts the fate towards Bolwig's organ formation [4]. *tll* knockdown in the optic lobe disrupts neurogenesis, producing defective optic ganglia [5].

Rpd3 is a maternally expressed histone deacetylase (HDAC) [6] that counteracts the spreading of heterochromatin [7]. An *Rpd3* null mutation enhances position effect variegation of the *white* gene in the *Drosophila* eye, promoting silencing which suggests that a wild-type *Rpd3* counteract silencing [8]. However, there have been no previous reports about the direct regulation of gap genes by the histone deacetylase, *Rpd3*. Tailless is regulated by a few well-established co-repressors, like Atrophin and Brakeless [9]. Atrophin (also known as Grunge; Gug) interacts with the histone deacetylase, HDAC1 [10,11], while Brakeless (also known as scribbler; sbb) interacts with Tailless via the DNA-binding domain [9]. Tailless is also regulated by factors like Ttk69 and Hsf [3]. Prospero interacts with Rpd3 to regulate the targeting of the dendrites of the olfactory projection neurons [12]. Polycomb protein, E(Z) and Rpd3 reportedly bind to polycomb response element (PRE) of *Ultrabithorax(Ubx)*, thereby deacetylating histones and silencing gene expression [13].

The EGFR or dEGFR (EGFR in *Drosophila*) activity in *Drosophila* initiates neurogenesis in the optic lobe to produce neuroblasts [14,15] and control the population and survival of neuroectodermal progenitor cells [16]. EGFR interacts with Jun N-terminal kinase-mitogen-activated protein kinase (JNK-MAP) kinase affecting the mushroom body development [17]. During early development, it has prominent roles of patterning the ventral epidermis [18] and providing survival signals to avert apoptosis [19]. The EGFR signalling is inhibited in the imaginal discs and eyes during early neural development by a human neural cell adhesion molecule (NCAM) orthologue, Fasciclin 2 (Fas2) [20]. EGFR is hyperactivated in the eye, notum and wing in loss-of-function mutants of *fas2* [20]. EGFR inhibition occurs when it is degraded by Anterior open (Aop), also known as Yan. When Fas2 is downregulated, it results in EGFR upregulation and thereby promotes cell proliferation. Alternatively, EGFR is inhibited when the EGFR ligand, Spitz fails to bind to EGFR as it is sequestered by Argos (Aos) [20]. Aos inhibits cellular differentiation while Anterior open triggers differentiation and division in embryos [21]. When Fas2 is downregulated, it leads to the Aop degradation and EGFR activation [20].

Here, we found a new role of *Rpd3* in regulating the expression of gap genes, especially *tailless*, analysed both quantitatively and phenotypically. The embryonic heteroallelic mutants for *Rpd3* showed a severe loss of Tailless expression. This finding was further supported by the increased enrichment of the heterochromatic markers in the promoter region of the *tailless*, in the case of the *Rpd3* heteroallelic mutants. Previous reports have shown HDACs responsible for the progression of neurodegenerative diseases. There are pieces of evidence of memory amelioration in mouse models [22] and polyglutamine-dependent neurodegeneration correction in *Drosophila* [23] upon HDAC inhibitor treatments. We, therefore, hypothesized that the *Rpd3* heteroallelic mutations change the expression of Tailless in the larval mutant brains. A significant decrease in the expression of Tailless was observed in the *Rpd3* heteroallelic mutant larval brains, similar to that in the embryos. Because Rpd3 has various interacting partners, we also attempted to find out whether the change in Tailless was owing to Rpd3 alone or owing to the interacting partners. We investigated the expression of Tailless in the larval brains for the mutants of *Rpd3* as well as the interacting partners of Rpd3 and Tailless. We showed that there was a severe decrease in Tailless in the interacting genotypes (both Rpd3 and the individual interacting partners) compared to the individual mutants. This signifies that Rpd3 was necessary and essential for the normal expression of Tailless.

Tailless, the primary readout for the study, reportedly co-express with Fas2 in the lamina precursor cells with a lateral high and medial low expression in the outer proliferation centres of the optic lobe [5]. Hence, we also attempted to study the expression of Fas2 in the mushroom bodies of the *Rpd3* heteroallelic mutants. In *Drosophila*, the mushroom bodies or corpora pedunculata constitute the seat of olfactory learning and memory [24,25]. The decrease in Tailless was found to be in unison with the decrease in Fas2 in the *Rpd3* heteroallelic mutant. Changes like decrease in embryo volume and alteration in the larval ventral epidermal patterning of the *Rpd3* mutant further justified the key role of the EGFR pathway in regulating early segmentation and brain development. This pioneering study thus highlights the importance of Rpd3 in the regulation of Tailless and establishes a significant insight that normal functioning of Rpd3 and Tailless are critical for the *Drosophila* early brain development under the influence of the EGFR pathway.

## 2. Material and methods

### 2.1. Fly strains and the genetic crosses

All the fly strains were reared with standard *Drosophila* culture media at the optimal temperature of 25°C. The exact genotype of each fly is described in the FLYBASE unless noted elsewhere. The genotypes of each fly strain are as follows: Canton-S (*CS*) (referred to as wild-type), *Rpd3(15-1)/TKG, Rpd3N(null)/TKG*, etc.

For further analysis, the different mutants of the interacting partners of Rpd3 and Tailless were procured from the Bloomington *Drosophila* Stock Center (BDSC), Indiana, having the BDSC numbers 12 772, 11 615, 12 350, 30 715, 4163, 5491, 5458, 1004 and 3101. The stocks, *Pc^XT109^* and *ph^del^* stocks, are null mutants (gift from Dr Giacomo Cavalli, Institute of Human Genetics, CNRS and the University of Montpellier, Montpellier, France).

Crosses were initially set between the *Rpd3N/TKG* and *Rpd3(15-1)/TKG* to obtain heteroallelic escaper mutants*.* The escaper mutants obtained from the cross between *Rpd3N/ TKG* and *Rpd3(15-1)/TKG* were selected by screening for non-GFP progenies. These escaper progenies were further crossed with the interacting partner allele, producing the resultant [*Rpd3N/Rpd3(15-1), interacting partner/*+] when the interacting locus is on the same chromosome as *Rpd3* or [*Rpd3N/Rpd3(15-1*); *interacting partner*/+] when the interacting locus is on the separate chromosome.

### 2.2. Embryo collection

The allele, *Rpd3 (15-1)*, is a loss-of-function allele with viable and fertile individuals [8,26]. The *Rpd3 null* allele or *Rpd3N* that was considered for this study is lethal in the larval stage when present in a homozygous condition [26]. To generate escaper *Rpd3* heteroallelic mutant, virgin females of the *Rpd3 null* mutation with green fluorescent protein (GFP) balancer marker, *Rpd3N/TKG* were crossed with an equal number of males of the genotype- *Rpd3(15-1)/TKG* carrying different *Rpd3* alleles in the embryos collection cages as cited by Wieschaus & Nüsslein-Volhard [27]. The embryos aged between 15 and 120 min after egg laying, from crossed parental flies were considered to be well synchronized. These eggs were harvested on the agar plates at 24°C. Embryos without a balancer marker (here, GFP for TKG) constituted the escaper populations and were selected under a ultraviolet (UV) binocular microscope. These embryos were collected as heteroallelic mutants between the two different alleles of *Rpd3*.

### 2.3. Fixation of the collected embryos

The *Rpd3* heteroallelic embryos without GFP expression (constituting the escaper mutants) were fixed in 50% hypochlorite solution for 4–5 min, which removed the outer chorionic layer and made the embryos afloat. These embryos were further rinsed in a solution of PBS-0.5% Triton X-100 and washed in distilled water two to three times. The embryos were then transferred to a 1.5 ml microfuge tube containing a 1 : 1 ratio of *n*-heptane/PEM (1 mM EGTA, 1 mM MgCl_2_ and 0.1 M PIPES)—formaldehyde and incubated for 15 min on a shaker. Later, the embryos were incubated in a fresh microfuge tube containing 500 µl of *n*-heptane and 500 µl of methanol. The tube was shaken vigorously for 40–50 s until the contents separated into two distinct phases. The upper heptane layer, which contains empty vitelline membranes, was carefully removed. The fixed embryos were used for immunostaining or stored at −20°C for future use, as described in [27].

### 2.4. Embryo immunostaining

An appropriate number of embryos, already fixed and preserved in methanol, were taken in new 1.5 ml microfuge tubes and the methanol was drained out from the tube. One millilitre of fresh methanol was added to it followed by 50 µl of 30% H_2_O_2_, with persistent rocking at room temperature for 20 min. The peroxide mixture was removed and 1 ml of 100% methanol was added to each tube followed by constant rocking at room temperature for 5 min, repeating at least three times. This solution was later replaced by adding 50% methanol with rocking at room temperature for 5 min. The methanol was then removed followed by four to five times rinsing with 1 ml PBT (1× phosphate buffered saline (PBS) with 0.3% Triton X-100) coupled with intermittent rocking at room temperature. The PBT was removed and 1 ml of a mixture of PBT with 2% anti-goat serum was added to each tube (for blocking) at room temperature with constant rocking for an hour. The primary antibodies against Bicoid, Hunchback, Kruppel, Giant and Tailless (from the Asian Distribution Centre for Segmentation Antibodies; 1 : 200) were diluted in the blocking solution. Embryos were transferred to 0.5 ml microfuge tubes. After removing the excess blocking solution, 75–150 µl of the primary antibodies was added to each tube, followed by incubation at room temperature for 1–2 h with persistent rocking at 4°C overnight. After overnight incubation, the primary antibodies were removed and the embryos were transferred to 1.5 ml tubes and rinsed for about eight times with 1 ml PBT (rocking at room temperature). The embryos were blocked with PBT and 2% anti-goat serum (AGS) at room temperature for 1 h with continuous rocking.

After the removal of the blocking solution, 75–150 µl of horseradish peroxidase (HRP)-conjugated secondary antibodies (Sigma Aldrich; 1 : 200) was added and incubated at room temperature for 45 min. Removing the secondary antibody, the embryos were rinsed about eight times with PBT. While the PBT rinsing was in progress, 3,3′-diaminobenzidine (DAB) solution was prepared, where two parts of the DAB solution were mixed with one part of 3% NiCl_2_ [28]. The embryos were treated with the above solution and rocked for about 10 min at room temperature. The intensity of staining was carefully observed and when the desired intensity was obtained, the reaction was stopped by the addition of PBT. The stained embryos were subjected to different increasing gradients of ethanol to dehydrate, followed by two changes of 100% ethanol after finally clearing in gradients of 50 and 100% Xylene. The immunostained embryos were mounted in the VECTASHIELD Mounting Medium (H-1000) and visualized under a light microscope.

### 2.5. Larval brain immunostaining

The third instar larvae from the various heteroallelic and interacting genotype mutants were screened under a UV filter-enabled binocular microscope and the brains were dissected in ice-cold 1× PBS. The tissues were fixed in 4% paraformaldehyde solution in PBS and incubated at room temperature for 20 min. The fixative was then removed and the tissues were rinsed in 0.3% PBT (0.3% Triton X-100 in 1× PBS) three times for 10 min each. The tissues were subsequently incubated in the blocking solution (2% AGS in PBT solution) at 4°C for 1 h. The tissues were then incubated in primary antibodies—mouse anti-Tailless (SantaCruz Biotechnology Inc., B-10; 1 : 50), rabbit anti-Asense (a gift from Dr Yun-Nung-Jan, Howard Hughes Medical Institute, UCSF; 1 : 5000), rabbit anti-Prospero (a gift from Dr Yun-Nung-Jan, Howard Hughes Medical Institute, UCSF; 1 : 1500), mouse anti-Fas2 (*Drosophila* Studies Hybridoma Bank (DSHB), 1D4; 1 : 50) and mouse anti-Repo (DSHB, 8D12; 1 : 50) overnight. The next day, the primary antibodies were drained off and the tissues were rinsed in PBT for three to five times at 4°C for 10 min each. The secondary antibodies, the donkey anti-rabbit and anti-mouse conjugated to Cyanine-3 (Cy-3) (Jackson Immuno-research Laboratories, Inc., 711–165–152, 715–165–150; 1 : 200), were dissolved in the blocking solution and incubated for 2 h at room temperature. After 2 h, the antibodies were drained off and the tissues were rinsed in PBT for 10 min each, three times. Then the tissues were rinsed in 1× PBS and mounted in the VECTASHIELD mounting media with DAPI (4', 6-diamidino-2-phenylindole) (H-1200). Images were captured using an Olympus FV1000 confocal microscope at lower (10× and 20×) as well as higher magnifications (40×), respectively. All the images are presented as *XY* figures as whole *Z*-stacks. All the images were adjusted and assembled in Adobe Photoshop CS6.

### 2.6. Western blot hybridization

Nearly 0.2 g embryos were collected in a 1.5 ml Eppendorf tube and homogenized thoroughly in the lysis buffer (50 mM Tris pH 8.0, 1 mM EDTA, 0.5% Triton X-100,1 mM PMSF and 1× protease inhibitor (Roche) (depending upon the number of embryos). This sample was centrifuged at 16 000 rcf at 4°C for 15 min. This supernatant was collected in a new 1.5 ml microfuge tube and stored at −20°C until further use. The protein concentration of the lysates (the supernatants collected previously) was measured using the Bradford assay and run on 10% SDS–PAGE gel. The protein bands were transferred onto the polyvinylidene difluoride (PVDF) membrane. The PVDF membranes were blocked with a 5% milk solution for 1 h, at room temperature. The blots were then incubated with primary antibodies (Bicoid, Kruppel, Giant, Hunchback and tailless; 1 : 500) and mouse Anti-β Actin antibody (Abcam, 8224; 1 : 2000) at 4°C overnight and then rinsed for 10 min, five times, with TBST buffer (Tris-buffered saline, 0.1% Tween 20) (1500 mM NaCl, 200 mM Tris, pH 7.5, 2% Tween-20). The blots were incubated with secondary antibodies conjugated to HRP for 1 h (Sigma Aldrich; 1 : 3000) at room temperature. Membranes were rinsed for about five times for 10 min each with the TBST buffer. Immunoreactive bands were detected by enhanced chemiluminescence (ECL) as per the protocol described [29].

For the western blot analysis from the heteroallelic and *trans*-heterozygotic larval brain, the total protein extracts from the larval brains were prepared in the lysis buffer (50 mM Tris pH 8.0, 1 mM EDTA, 0.5% Triton X-100, 1 mM PMSF and 1× protease inhibitor (Roche) and analysed as described. The primary antibodies, mouse anti-dEGFR (Sigma Aldrich, E2906; 1 : 1000), mouse anti-Tailless (SantaCruz Biotechnology Inc., B-10; 1 : 500), rabbit anti-Asense (a gift from Dr Yun-Nung-Jan, Howard Hughes Medical Institute, UCSF; 1 : 500), rabbit anti-Prospero (a gift from Dr Yun-Nung-Jan, Howard Hughes Medical Institute, UCSF; 1 : 1000), mouse anti-Fas2 (*Drosophila* Studies Hybridoma Bank (DSHB), 1D4; 1 : 500), mouse anti-Repo (DSHB, 8D12; 1 : 500), mouse Anti-beta Actin antibody (Abcam, 8224; 1 : 2000) and secondary antibodies conjugated to HRP (Sigma Aldrich; 1 : 3000) were used, respectively. Immunoreactive bands were detected by enhanced ECL and the densitometries of the bands were performed using ImageJ software (NIH, USA). The mean values from three independent experiments were represented with error bars corresponding to ±s.e.m. as indicated. Student's *t*-test was performed between the wild-type and mutant groups. Statistical analyses were performed and the graphical illustrations were processed using Microsoft Excel 2007 and GraphPad Prism 8 software. The statistical significance for each set of data has been indicated as ***p ≤ 0.001, **p ≤ 0.01 and *p ≤ 0.05, respectively.

### 2.7. Chromatin immunoprecipitation

To examine the role of the epigenetic modifiers in the regulation of *tailless*, the promoter sequences were predicted (since unavailable in the NCBI gene) using a promoter prediction tool, Neural Network Promoter Prediction, developed by the Berkley *Drosophila* Genome project. Accordingly, the forward and the reverse primers were designed (figure 2*a*) and the chromatin was isolated for the heteroallelic mutant of *Rpd3*. The respective DNA was immunoprecipitated against various euchromatic and heterochromatic markers like the H3K9me3, H3K27me3, RNA Pol II, H3K9Ac and H3K4Ac. The immunoprecipitated samples were subjected to a real-time quantitative reverse transcription-polymerase chain reaction (RT-qPCR) to analyse the real-time amplification of the genes for the mutant phenotype after comparison with the 18S rRNA. The data were analysed and the normalized values were plotted on bar graphs to allow genotypewise comparison. The ChIP was performed as per the protocol reported earlier [30,31]. Briefly, 150–200 µl of *Drosophila* embryos (kept frozen in 1× PBS at −80°C) was thawed and strained in 10 ml homogenization tubes. To it, 5 ml of buffer A1 (60 mM KCl, 15 mM NaCl, 4 mM MgCl_2_,15 mM HEPES (pH 7.6), 0.5% Triton X-100, 0.5 mM DTT, 10 mM sodium butyrate, protease inhibitor, Roche (25×)) was added and homogenized followed by centrifugation at 400*g* for 1 min. The supernatant collected thereafter was re-centrifuged at 1100*g* for 10 min at 4°C, to obtain a pellet which was resuspended in 5 ml PBS and formaldehyde was added to it to a final concentration of 1% and mixed well. Cross-linking was done at room temperature for 10 min and was quenched by adding glycine to a final concentration of 225 mM. The homogenate was pelleted down at 4000 rpm for 15 min at 4°C. The pellet was washed twice in cold PBS containing 1× protease inhibitor cocktail and finally resuspended in buffer A (5 mM PIPES pH8.0, 85 mM KCl, 1× protease inhibitor cocktail (Roche), 50 U ml^−1^ SUPERNase) with 0.5% NP-40 and incubated on ice for 10 min. The crude fraction was pelleted by centrifugation at 5000 rpm for 5 min at 4°C and the pellet was washed once with buffer A, resuspended in buffer B (1% SDS, 10 mM EDTA, 50 mM Tris–HCl pH 8.1, 1× Roche protease inhibitor cocktail, 50 U ml^−1^ SUPERNase) and incubated on ice for 10 min. Lysates were sonicated for 15 cycles (30 s-on 30 s-off) in a Bioruptor^®^ Plus sonication device (Diagenode SA) to achieve chromatin fragments. The sonicated chromatin was diluted with the ChIP dilution buffer (0.01% SDS, 1.1% Triton X-100, 1.2 mM EDTA, 16.7 mM Tris–HCl pH8.1, 167 mM NaCl, 1× protease inhibitor cocktail (Roche)) and pre-cleared with Protein A-Sepharose beads (GE Healthcare; 17-0780) for 1 h at 4°C. About 200 µg of pre-cleared chromatin was incubated with the antibodies: rabbit anti-H3K9me3 (Upstate, 07-442; 1 : 1000), rabbit anti-H3K27me3 (Upstate, 07-449; 1 : 1000), mouse anti-RNA Polymerase II CTD repeat YSPTSPS (Abcam, ab817; 1 : 1000), rabbit anti-H3K9Ac (Upstate, 06-942; 1 : 1000) and rabbit anti-H3K4Ac (Upstate, 07-539; 1 : 1000) overnight and the samples were incubated with Protein A-Sepharose beads for immunoprecipitation. The immunoprecipitated complexes were washed sequentially in low salt wash buffer (0.1% SDS, 1% Triton X-100, 2 mM EDTA, 20 mM Tris–HCl pH8.1, 150 mM NaCl), high salt wash buffer (0.1% SDS, 1% Triton X-100, 2 mM EDTA, 20 mM Tris–HCl pH 8.1, 500 mM NaCl), LiCl wash buffer (0.25 M LiCl, 1% NP-40, 1% Deoxycholate, 1 mM EDTA, 10 mM Tris–HCl pH8.1) and TE buffer (10 mM Tris–HCl, 1 mM EDTA pH 8.1). The nucleic acid was eluted with elution buffer (1% SDS, 0.1 M Na_2_HCO_3_) and cross-links were reversed for 2 h at 65°C in the presence of 200 mM NaCl. Chromatin samples were treated with Proteinase-K and RNA was isolated using the TRIZOL reagent (Invitrogen) following the manufacturer's protocol (Promega). Genomic DNA contamination was removed from immunoprecipitated RNA samples using the TURBO DNA-free kit (Ambion). The samples were amplified following a real-time PCR protocol using specific primers. The sequences of the promoter amplification added were as given below.

### 



TII_pro: forward primer: 5′-TGCTGCGTTCCTTGCTCAATG-3′,

reverse primer: 5′-TCGCTGAGATCACGATGGGTT-3′.

### 



18S rRNA: forward primer: 5′-CCTTATGGGACGTGTGCTTT-3′,

reverse primer: 5′-CCTGCTGCCTTCCTTAGATG -3′.

### 2.8. Reverse transcription–polymerase chain reaction (PCR) and real-time quantitative reverse transcription-PCR

The total RNA was extracted from *Drosophila* embryos by the Trizol method (Invitrogen) and purified by using the RNeasy mini kit (Qiagen). RNA was treated with DNase I (Ambion) for 30 min at 37°C. cDNA was synthesized using the Superscript™ II RT-PCR kit (Invitrogen). Strand-specific reverse transcription–PCR (RT-PCR) was done using the 18S rRNA and other gap gene promoter primer sets. PCR amplification was performed using AmpliTaq Gold system (Promega). Quantitative RT-PCR was carried out using the LightCycler^®^ 480 System (the Roche Applied Science). The KAPA SYBR^®^ FAST qPCR Master Mix (2×) Kit (KAPA Biosystems) was used according to the manufacturer's directions. Transcript levels were normalized to 18S rRNA levels. The data were graphically represented as the relative enrichment of the epigenetic markers after comparison with the 18S rRNA and the level of statistical significance for each set of data has been indicated as ratio ± s.e.m of three independent experiments.

The *Rpd3* heteroallelic larval brains were collected and total RNA was isolated by the Trizol method (Invitrogen). The RNA was purified using the RNeasy mini kit (Qiagen), followed by treatment with DNase1 (Ambion) for 30 min at 37°C. cDNA was synthesized by RT–PCR using the RNA to cDNA EcoDry™ Premix (Oligo dT) (Clontech). Quantitative RT-PCR was run on a CFX96™ Real-Time System with the C1000 Touch™ Thermal Cycler (Bio-Rad) with each PCR mixture containing a 0.5 µl cDNA template and 10 nM primers in 20 µl of KAPA SYBR^®^ FAST qPCR Master Mix (2×) (KAPA Biosystems). The analyses were done using the Bio-Rad CFX Maestro™ Software. Expression values were normalized to those obtained with the control 18S rRNA. The mRNA was measured using the primers listed as follows:

*tll:* forward primer*:* (*5′-ATGAATTTCGCCCAGCTGCT-3′*)*,* reverse primer*:* (*5′-AGCCCTCAGACACTCGTACT-3′*)*; Rpd3:* forward primer*:* (*5′-TTAAGCTGCACATTAGTCCCAGC-3′*)*,* reverse primer*:* (*5′-CTCGGGAATCGCTTGGATTTGAA-3′*); *18S rRNA:* forward primer*:* (*5′-TTGTGCTGAAGAAGGCCGAT-3′*)*,* reverse primer*:* (*5′-CTGCCTGTTGAGGAACCAGT-3′*).

The data were graphically represented as the relative mRNA fold change compared to the 18S rRNA and the level of statistical significance for each set of data has been indicated as mean ± s.e.m. of three independent experiments (*t*-test) ***p ≤ 0.001, **p ≤ 0.01 and *p ≤ 0.05 respectively.

### 2.9. The scanning electron microscope imaging of the larvae

*Drosophila* third instar larvae for the wild-type (*Canton-S*) and the heteroallelic *Rpd3* mutant were collected from the food bottles pre-incubated at 18°C, rinsed in 1× PBS and affixed on the stubs coated with double-sided tape. They were subjected to gold sputtering under the plasma pressure for 15 s and were imaged under the scanning electron microscope (model no. 3400N, Hitachi, Japan) at 5.0 kV acceleration speed and 27× and 150× magnification.

## 3. Results

The gene *Rpd3* (*Reduced potassium dependency 3*) encodes the class I histone deacetylase which is a mammalian homologue of the HDAC1 and HDAC2 [8,32,33]. When *Rpd3* is knocked down, there is a global increase in telomeric H3 and H4 histone acetylation on the polytene chromosomes [34]. The present study presented a new role of *Rpd3* in regulating the expression of gap genes in the embryos and also demonstrated how particular mutations in *Rpd3* and its interacting partners affect Tailless expression in the larval brain. To investigate the involvement of the Rpd3, we have used two *Rpd3* loss-of-function alleles, *Rpd3N* and *Rpd3(15-1)*, in heteroallelic combinations.

**Figure 1.**
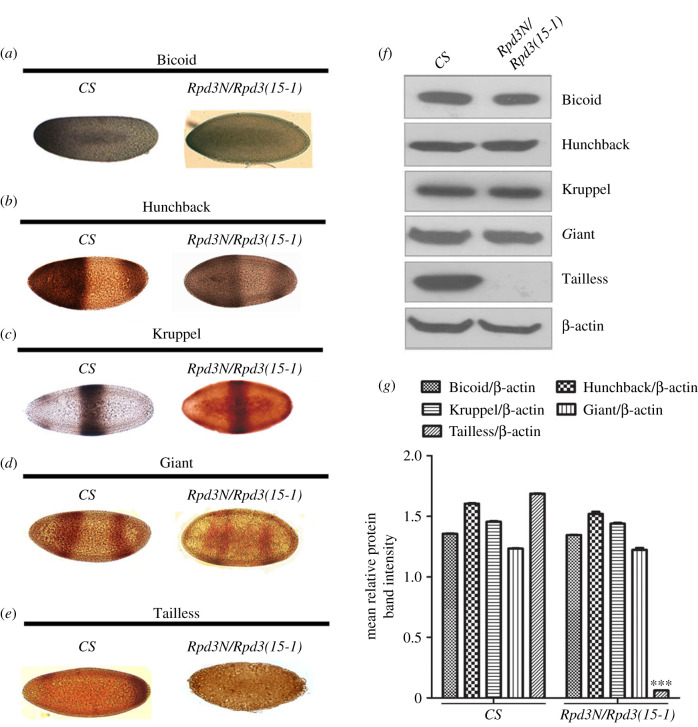
The *Rpd3* mutation results in the significant modulation of the different gap proteins but negligibly affects the expression of the maternal gene, *Bicoid*. The *Rpd3* heteroallelic mutant embryos aged about 2 h after egg laying (AEL) were collected and the embryos without GFP balancer marker, representing the escaper populations were selected. These embryos were stained for the localization of (*a*) Bicoid, (*b*) Hunchback, (*c*) Kruppel, (*d*) Giant and (*e*) Tailless (brown, HRP-DAB precipitate), relative to the wild-type (*CS*) and visualized under a light microscope. (*f*) Western blots depicting the quantitative estimation of the different gap genes, *hunchback, Kruppel, giant* and *tailless,* and the maternal effect gene, *bicoid*, in the heteroallelic *Rpd3* mutant compared to the wild-type (*CS*)*.* The lower panel shows the relative amount of β-actin in the *Rpd3* mutant embryos. Data represent the mean ± s.e.m. from three independent experiments. (*g*) Graphical representation of the quantitative western blot hydbridization for the genes, *hunchback, Kruppel, giant* and *tailless*, and the maternal effect gene, *bicoid*, in the heteroallelic *Rpd3* mutant compared to the wild-type (*CS*)*.* The data are a representation of three independent experiments and depict the mean ± s.e.m. with ***p ≤ 0.001 (*n* = 3).

In *Drosophila melanogaster,* the *Rpd3* gene is cytologically located in the 64C1-2 region of chromosome 3. The *Rpd3(15-1)* allele has a P-element insertion of 1.8 kb from the 5' end of the *Rpd3* transcript in the putative regulatory region [8]. The *Rpd3(15-1)* loss-of-function allele is viable and fertile [26]. *Rpd3N* is a null allele, produced by a P-element excision and deletion of approximately 870 bp in *rpd3^04556^* in the 5′-coding region of the gene [26]. The expression of gap genes in the *Rpd3* heteroallelic mutant embryos was analysed via immunostaining and quantitative western blot hybridization. The maternal gene, *bicoid (bcd)*, produces a morphogen gradient that dictates the expression of subsequent gap genes in the developing embryos [35]. When the early, age-synchronized heteroallelic *Rpd3* mutant embryos were immunostained with the Bicoid antibody, there was no change in the intensity of Bicoid (figure 1*a*) similar to the expression observed in the quantitative western blot (figure 1*f*,*g*). Thus, heteroallelic mutations in *Rpd3* embryos do not affect the expression of Bicoid.

### 3.1. *Rpd3* loss-of-function does not modulate Hunchback, Kruppel or Giant expression

The gene, *hunchback*, regulates the trunk formation in *Drosophila* by establishing an anterior–posterior gradient from the unfertilized egg to the developing zygote [36]. When the *Rpd3* heteroallelic mutant embryos were immunostained with Hunchback (Hb) antibody (figure 1*b*), and the expression was further substantiated by a western blot (figure 1*f*,*g***)**, the expression of Hb did not change, compared to the wild-type. Therefore, *Rpd3* does not regulate the expression of Hunchback.

*Krüppel* expresses in the early stages of the embryos in the form of a central band [37]. In the age-synchronized embryos of *Rpd3* heteroallelic mutants, Kruppel showed no change in expression compared to the wild-type, in immunostaining (figure 1*c*) as well as western blot (figure 1*f*,*g*). Hence, *Rpd3* does not regulate Kruppel expression.

Giant (gt) expresses in the early embryos producing two broad stripes with a bare central mid-region [38]. When age-synchronized *Rpd3* heteroallelic embryos were immunostained with Giant, the expression of Giant showed no change in the mutant compared to the wild-type (figure 1*d*). The western blot also substantiated the above result (figure 1*f*,*g*) and concluded that *Rpd3* heteroallelic mutation does not affect the expression of Giant.

### 3.2. Rpd3 regulates Tailless expression in *Drosophila* embryo

*Tailless(tll)* defines the posterior boundary of expression for *hb* and *gt* [39] and regulates the development of the head and posterior embryonic regions. When the same-aged *Rpd3* heteroallelic mutant embryos were immunostained, there was a severe loss of Tll expression in the *Rpd3* heteroallelic mutant compared to the wild-type (figure 1*e*). The result of the western hybridization also substantiated the complete loss of Tailless expression in the *Rpd3* heteroallelic mutant embryos (figure 1*f*,*g*), showing that Rpd3 regulates Tailless in *Drosophila* embryos.

Depending upon the previous evidence of Rpd3 in brain development, we focused on our subsequent studies to understand how Tailless decreased such severely in the heteroallelic *Rpd3* mutants. It led to a proposition that Rpd3 might modulate the expression of gap genes through epigenetic regulation, highlighting the importance of a functional Rpd3-dependent chromatin complex for *tll* regulation and anterior–posterior axis formation.

### 3.3. The Rpd3 complex epigenetically regulates Tailless expression

**Figure 2.**
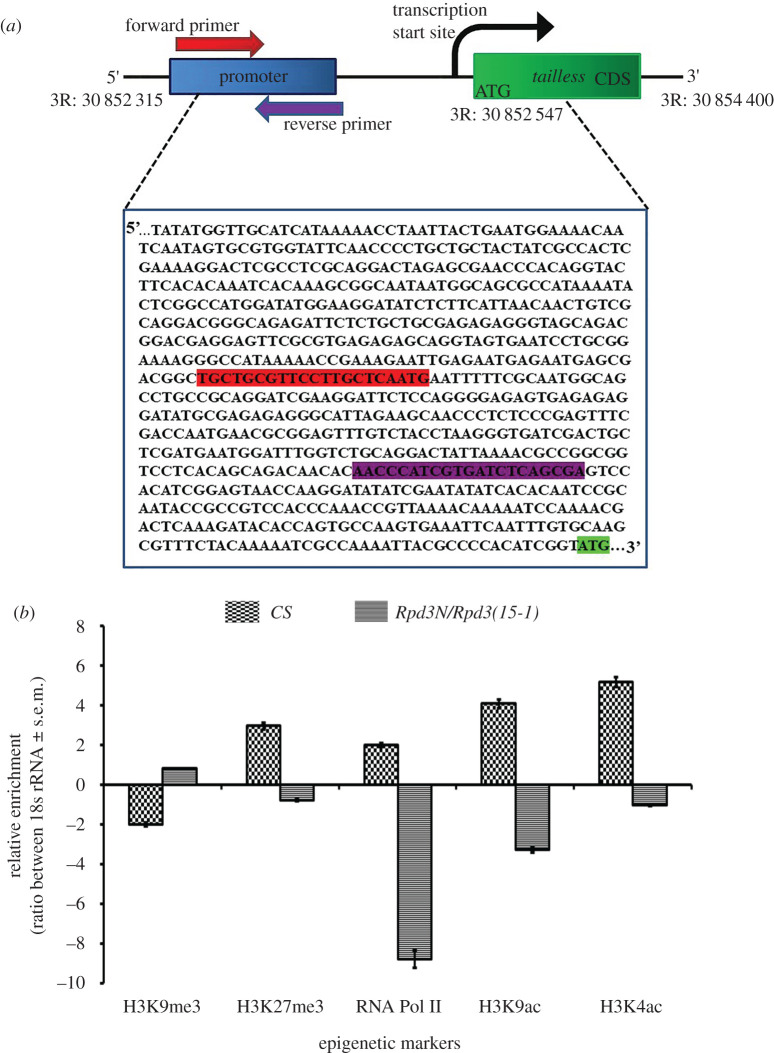
(*a*) The schematic of the relationship of transcription start site (TSS) and the primer annealing sites for the gene, *tailless*, used in ChIP assay. The promoter-based primers were designed for the gene, *tailless,* located on the chromosome 3R in *D. melanogaster.* The gene sequence shown in the box represents those that are upstream to the coding region of *tailless*. The 5′ untranslated region (UTR) of *tailless* starts from 30 852 315 nucleotide (nt) and the 3′ UTR ends at 30 854 400 nt. The CDS of *tailless* begins from 30 852 547 nt with the start codon ATG. The sequence annealing with the forward primer: 5′-TGCTGCGTTCCTTGCTCAATG-3′ has been marked in red and those that anneal with the reverse primer: 5′-TCGCTGAGATCACGATGGGTT-3′ have been shown in purple. The CDS of *tailless*, beginning with the start codon, ATG has been highlighted in green. (*b*) The ChIP assay data showing the relative enrichment of the epigenetic markers on the promoter of *tailless* in the *Rpd3* heteroallelic mutant. The ChIP assay was performed to assess the relative enrichment of the epigenetic markers in the promoter region of *tailless*. The age-synchronized, 2 h after egg laying (AEL) embryos from the wild-type (*CS*) as well as *Rpd3* heteroallelic mutants were collected and the DNA was isolated. It was immunoprecipitated with various markers for heterochromatin formation like H3K9me3 and H3K27me3, and markers for euchromatin formation like H3K9ac and H3K4ac. RNA Pol II which acts as a marker depicting transcriptional activity was also assessed. The relative enrichment of these markers on the *tailless* promoter was assessed using the real-time PCR, after comparison with the 18s rRNA for both the wild-type (*CS*) and *Rpd3* heteroallelic mutant. Each of the markers has been plotted in the graph and the trends in the variation of these markers represented with error bars corresponding to s.e.m. from three independent experiments.

The results obtained previously have led to the proposition that changes in Tailless in the *Rpd3* heteroallelic mutant were possibly owing to the regulators of the transcriptional machinery. To confirm the involvement of histone-deacetylase associated with Tailless expression in the *Rpd3* mutant, an *in vivo* chromatin immunoprecipitation (ChIP) assay based on the *tailless* promoter was performed (figure 2*a*). This study assesses the capacity of each factor of the transcriptional machinery and associated histone tail modifiers to bind with early recognized promoter *in vivo* in the chromatin [40,41]. The underlying principle states that promoters which have binding sites for the transcriptional machinery undergo hindrance owing to the chromatin organization under specific cellular conditions, and hence, the transcriptional factors fail to interact (for details, refer to Material and methods). The occupancy of the histone modifiers (H3K9me3, H3K27me3, RNA PolII, H3K9ac and H3K4ac) was assessed in the promoter region of *tailless* for the heteroallelic *Rpd3* mutants. Previous reports state that the repressive chromatin marks—H3K9me3 localize in 20 stable domains at the centromere [42] while H3K27me3 regulates homeotic genes, stem cell differentiation and regulation of development in vertebrates [43]. The stable H3K27me3 domains in *Drosophila* embryos and tissue culture are enriched for genes involved in development, transcription and segmentation [44]. H3K9ac associate with the transcription start sites (TSSs), corresponding to positive gene expression levels [45]. When histones are hyper-acetylated, Pol II starts transcription in the neuroectoderm [46]. Genome-wide ChIP analysis state that in the promoters of actively transcribed genes, H3K4ac localizes upstream to H3K4me3 [47,48]. H3K9ac localizes in the promoter regions of actively transcribed genes and stimulates transcription by recruiting transcription Factor IID (TFIID) [49], while the H3K9me2 and H3K9me3 marks are enriched in the pericentric heterochromatin structures to repress transcription [50,51]. In *Drosophila* embryos, until cycle 14a, the histone methylation marks, H3K4me1, H3K4me3, H3K36me3 and H3K27me3, and the acetylation mark, H3K9ac, are absent until transcription of the zygotic genes begins. The polycomb-associated mark, H3K27me3, regulates cell-specific silencing of developmental genes [52]; they are completely absent in maternal genes and enriched in the upstream and downstream promoter regions of the cycle 14 embryos [31,53,54].

ChIP assay for *Rpd3* heteroallelic mutant compared to the wild-type revealed a significant decrease in *tailless.* In the wild-type, *tailless* had an increased enrichment of the euchromatic markers, indicating an active state of the *tll* gene (figure 2*b*). In the *Rpd3* mutants, *tailless* had decreased enrichments of H3K9ac, H3K4ac, Pol II and H3K27me3 and an increased enrichment of H3K9me3 at the promoter, compared to the wild-type (figure 2*b*). Thus, the data established that a heteroallelic loss-of-function of *Rpd3* in the embryos, leads to the loss of Tailless owing to the decreased enrichment of H3K9ac, H3K4ac, H3K27me3 and Pol II.

### 3.4. The Tailless expression is reduced in the heteroallelic *Rpd3* mutant larval brains

**Figure 3.**
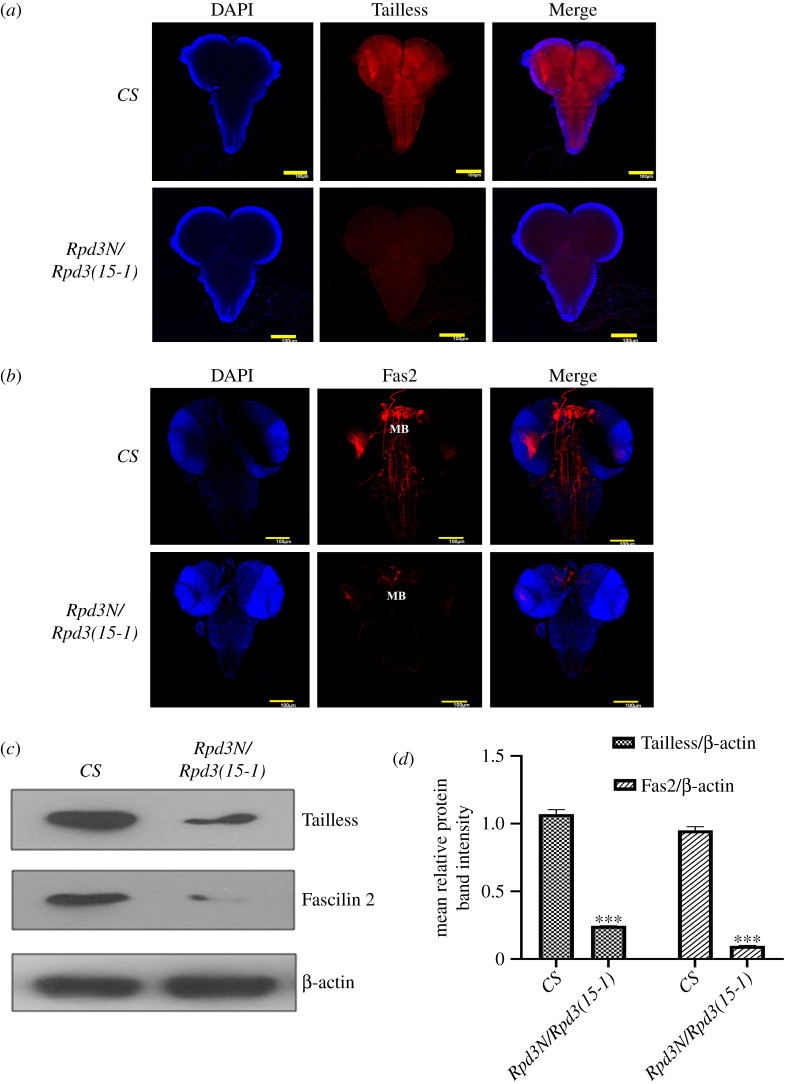
Alteration in Tailless and Fas2 expression in the *Rpd3* heteroallelic larval brains: (*a*) the late third instar heteroallelic *Rpd3* mutant larval brains were dissected and immunostained with Tailless (in red) to find out the localization in the larval brains. The images of the brains are all *Z*-projections of the *XY* plane. There was a reduction in the Tailless expression in *Rpd3* heteroallelic larval brains compared to the wild-type (*CS*). (*b*) The *Rpd3* heteroallelic late third instar larval brains were immunolabelled with the *Drosophila* NCAM homologue, Fas2, which localize in the mushroom bodies (MB) in the larval brain (red) and are *Z*-projections of the *XY* plane. A reduction in expression of Fas2 was observed in the *Rpd3* heteroallelic mutant larval brains. Scale bar, 100 µm. (*c*,*d*) The quantitative estimation of Tailless and Fas2 in the heteroallelic *Rpd3* mutant larval brains was performed by western blotting and normalized with β-actin. The data have been represented as the mean ± s.e.m. from three independent experiments. There was a decrease in the expression of Tailless and Fas2 compared to the wild-type (*CS*), which was also graphically illustrated. The mean relative protein band intensity was normalized with the internal control, β-actin (in the lower panel) band intensity, and represented with error bars corresponding to s.e.m. from three independent experiments in triplicate; ***p ≤ 0.001 (*n* = 3).

Former results have established the importance of Rpd3 in the Tailless regulation with an evident decrease in the heteroallelic *Rpd3* mutant. In the optic lobe neuroblast of the third instar larvae [55,56], Tailless ensures an optimum proliferation and prolonged maintenance of the mushroom body neuroblasts and ganglion mother cells [57]. Hence, *tailless* knockdown leads to defective optic lobes neurogenesis [5]. To investigate the possible role of Tailless in the *Rpd3* mutant on larval brain development, the heteroallelic *Rpd3N/Rpd3(15-1)* mutant third instar larval brains were immunostained with the Tailless antibody, where Tailless was significantly decreased in the mutant compared to the wild-type (figure 3*a*,*c*,*d*). When the immunostained *Rpd3* heteroallelic mutant larval brains were visualized under higher magnification, there was a significant reduction in the Tailless localization, particularly in the outer proliferation centre (OPC) and inner proliferation centre (IPC) regions, compared to that of the wild-type (electronic supplementary material, figure S1A). These findings confirm that Rpd3 has a critical role in the regulation of Tailless expression in the larval brain.

**Figure 4.**
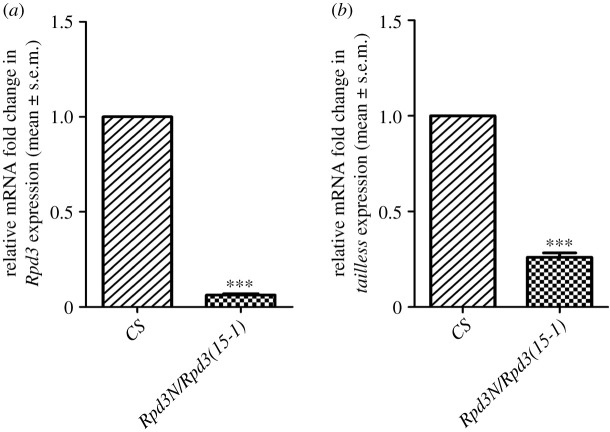
(*a*,*b*) Analysis of the expression of *Rpd3* and *tailless* mRNA in the third instar larval brain by the real-time qPCR. The third instar larval brains of the wild-type and heteroallelic *Rpd3* mutants were dissected and the total RNA was isolated from these samples. The cDNA was synthesized from the total RNA and subjected to a quantitative real-time PCR (qPCR). (*a*) The relative mRNA expression of *Rpd3* was measured, after normalization to 18S rRNA and represented as relative mRNA fold change (mean ± s.e.m.). There was a significant downregulation in the expression of *Rpd3* in the heteroallelic *Rpd3* mutants [*Rpd3N/ Rpd3 (15-1)*] compared to the wild-type (*CS*) (***p ≤ 0.001). The error bars correspond to standard error (s.e.m.) from three independent experiments in triplicate. (*b*) The relative mRNA fold change in the expression of *tailless* was measured after normalization to the 18S rRNA, represented as mean ± s.e.m. There was a significant downregulation in the mRNA expression of *tailless* in the heteroallelic *Rpd3* mutants compared to the wild-type (*CS*) (***p ≤ 0.001). The error bars correspond to standard error (s.e.m.) from three biological replicates.

The altered enrichment of the epigenetic modifiers in the promoter regions of *tailless* in the *Rpd3* mutant embryos suggested the further investigation of changes in *Rpd3* and *tailless* mRNA expression in the heteroallelic *Rpd3* third instar larval brains. The third instar larval mutant brains were dissected, the RNA was isolated and qPCR was performed after cDNA synthesis (figure 4*a*,*b*). There was a significant reduction in the relative expressions of both *Rpd3* (figure 4*a*) and *tll* mRNAs in the heteroallelic *Rpd3* third instar larval brain compared to the wild-type (*CS*) (figure 4*b*). This confirmed that the heteroallelic loss-of-function mutation of *Rpd3* in the larval brain leads to a decrease in the expression of *tailless* mRNA.

### 3.5. Rpd3 with its interacting partners regulates the expression of Tailless

**Figure 5.**
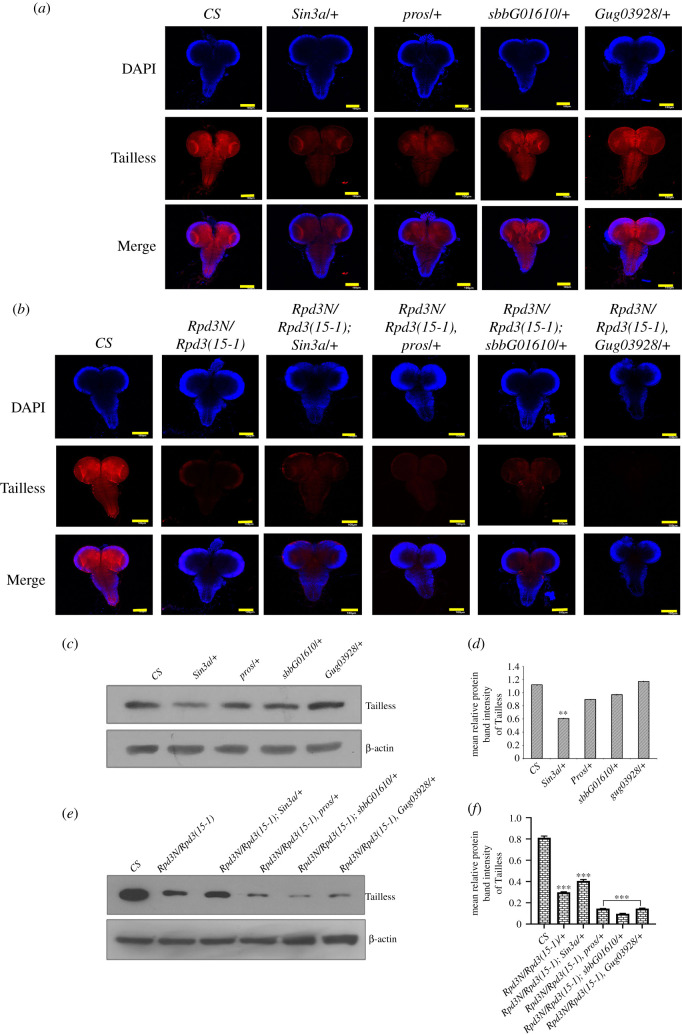
The variation in Tailless expression in the mutant brain of the interacting partner of Rpd3 and Tailless: (*a*,*b*) Rpd3 has reported interacting partners like Sin3a, prospero, scribbler and Atrophin. Crosses were set to obtain interacting genotype mutants between *Rpd3* and these interacting partner mutants. The interacting genotypes and individual mutant late third instar larval brains were immunostained with the Tailless antibody (in red) to find out a variation in Tailless expression. The images of the brain are all procured from *Z*-projections of confocal sections. There was a significant alteration in Tailless expression in the interacting partner mutants as well as in the interacting genotypes of these partners with *Rpd3*. Scale bar, 100 µm. (*c*,*d*) The western blot depict the quantitative estimation of Tailless in the different interacting partner mutants for Sin3a, prospero, scribbler and Atrophin, normalized with β-actin (in the lower panel). The immunoreactive bands are quantitated and expressed graphically as the mean relative intensity of the protein band density along with error bars corresponding to s.e.m. (**p ≤ 0.01). The data have been obtained from three independent experiments. (*e*,*f*) The western blot depict the quantitative estimation of Tailless in the different interacting genotypes, normalized with β-actin (in the lower panel). The immunoreactive bands are quantitated and expressed graphically as the mean relative intensity of the protein band density along with error bars corresponding to s.e.m. (***p ≤ 0.001). The data have been obtained from three independent experiments.

The previous result showed a significant reduction in the expression of Tailless in the heteroallelic *Rpd3* mutants. Therefore, the subsequent study aimed at investigating whether the change in Tailless was owing to Rpd3 itself or due to the interacting partners of Rpd3 and Tailless. We have immunostained the mutant larval brain to investigate Tailless expression in the interacting partner mutants as well as the interacting genotypes (mutated *Rpd3* as well as the mutated interacting partners) (figure 5*a*).

Sin3a is an Rpd3 co-repressor that forms scaffolds for the assembly of the HDAC complex [58,59], while Prospero interacts with Rpd3 and regulates terminal axon branching [12]. We found that in the *Sin3a*/+ and *prospero*/+ mutants, as well as in the interacting genotypes (*Rpd3N*/*Rpd3(15-1); Sin3a/+*) or (*Rpd3N*/*Rpd3(15-1), pros/+*), the expression of Tailless had reduced compared to the wild-type (figure 5*a*,*b*). In the interacting genotype (*Rpd3N*/*Rpd3(15-1), Sin3a/+*), Tailless was further reduced compared to the wild-type or the *Sin3a*/+ mutant alone. The *scribbler* gene (also known as *brakeless*) genetically interacts with *tll* and needs to be present as in the wild-type for initiating the repressive activity of Tailless [9]. Tailless expression was almost close to that of the wild-type in the *sbbG01610/+* mutant, but in the interacting genotypes (*Rpd3N/Rpd3(15-1); sbbG01610/+)*, there was a distinct reduction compared to the wild-type (figure 5*a–f*). Tailless interacts with Atrophin to repress the expression of Knirp [11]. When the larval brain for *Atrophin* mutants was immunostained, there was a negligible decrease in Tailless compared to the wild-type (figure 5*a*), but (*Rpd3N/Rpd3(15-1)*, *Gug03928/+*) showed a significant decrease (figure 5*b*,*e*,*f*).

Rpd3 executes gene silencing, by binding with the Polycomb protein at the polycomb responsive element (PRE) of the *Ultrabithorax* gene [13]. The Polycomb repressive complex 1 (PRC1) comprises Polycomb (PC), Posterior sex combs (PSC), polyhomeotic (PH) and Sex comb on midleg (SCM). PC associates with extra sexcombs (ESC) in a complex containing Enhancer of zeste (EZ), pleiohomeotic (PHO), polyhomeotic (PH), GAGA and Rpd3. Both the ESC and PC are crucial for polycomb-dependent gene silencing [60]. Tailless expression decreased only in the interacting genotype *(Rpd3N/Rpd3(15-1), Pc*/+) (electronic supplementary material, figure S2A,B). The Tailless expression was further substantiated by western blot (figure 5*c–f*) which concluded that the expression of Rpd3 is more crucial than the interacting partners for maintaining a normal Tailless expression in the larval brain.

**Figure 6.**
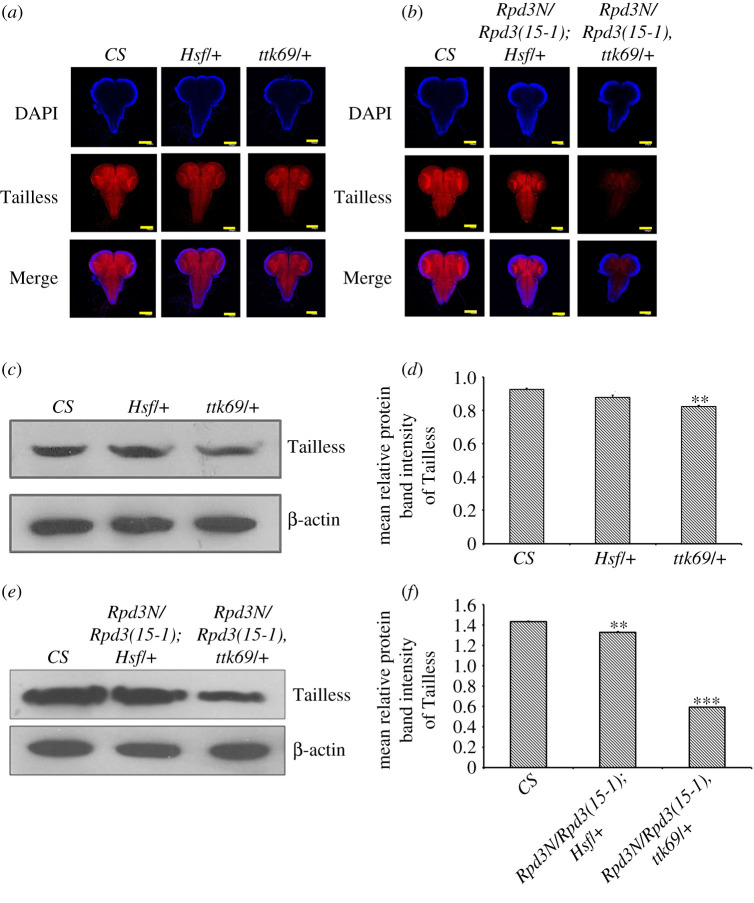
The variation in the expression of Tailless in interacting genotypes for *Rpd3* along with *Hsf* and *ttk69*. (*a*,*b*) Tailless is under the control of the modulators—Heat shock factor (Hsf) and Tramtrack69 (Ttk69). Interacting genotype mutants were obtained from crosses set between *Rpd3* heteroallelic progeny and the interacting partners, *Hsf* and *ttk69*. The interacting genotype late third instar larval brains were stained with Tailless (in red) to assess the localization of Tailless in these mutant brains and procured from *Z*-projections using a confocal microscope. There was a loss of expression of Tailless in the interacting genotypes (*Rpd3N/Rpd3(15-1)*, *ttk69/+*). When *Hsf* alone is mutated, there is no significant variation in the expression of Tailless. Scale bar, 100 µm. (*c*,*d*) The expression of Tailless was quantified in the case of the mutant larval brains of the individual interacting partners, *Hsf/+* and *ttk69/+* using the western blot and represented graphically. The mean relative protein intensity was obtained after normalization with β-actin (in the lower panel) from three independent experiments with three technical replicates each and represented with error bars corresponding to s.e.m.; **p ≤ 0.01 (*n* = 3). (*e*,*f*) The expression of Tailless was quantified in the case of the larval brains of the interacting genotypes (*Rpd3N/Rpd3(15-1); Hsf/+*) and (*Rpd3N/Rpd3(15-1), ttk69/+*) using the western blot hybridization and represented graphically. The mean relative protein intensity was obtained after normalization with β-actin (in the lower panel) from three independent experiments with three technical replicates each and represented with error bars corresponding to s.e.m.; ***p ≤ 0.001 and **p ≤ 0.01 (*n* = 3).

Tailless is regulated by factors like Ttk69 and Hsf. Ttk69 is a transcriptional repressor binding to tor-RE to repress Tailless [61], while Hsf, when activated, activates Tailless expression. The factors comprising Hsf and ttk69, when binding to GAF, alight on the tor-RE to repress *tll* [61]. The tailless expression did not decrease in the *Hsf/+* mutants and interacting genotypes (*Rpd3N/Rpd3(15-1); Hsf/+*) (figure 6*a*–*f*) but significantly decreased in (*Rpd3N/Rpd3(15-1), ttk69/+*) (figure 6*a*–*f*).

### 3.6. Fascilin2 expression, like Tailless, is regulated by *Rpd3* and its interacting partners

Fas2 is an inhibitor of EGFR signalling in *Drosophila* brain development [20]. Fas2 co-express with Tailless in the lamina precursor cells of the optic lobe and shows a high and medial low domain of expression in the developing outer proliferation centres [5]. It expresses in the cells forming the primordia for the mushroom body peduncle and medial lobe in the α/β and γ axon lobes [57]. To investigate whether the expression of Fas2 in the third instar larval brain of the heteroallelic *Rpd3N/Rpd3(15-1)* mutant correlated with Tailless, the Fas2 expression was studied in the mushroom body of the *Rpd3* heteroallelic mutant larval brains. The Fas2 expression in the *Rpd3* heteroallelic mutant brain decreased compared to the wild-type (figure 3*b–d*). At a higher magnification, the mushroom bodies of the *Rpd3* heteroallelic mutants showed a significant decrease in Fas2 with poorly developed lobes, compared to the wild-type (electronic supplementary material, figure S1B).

**Figure 7.**
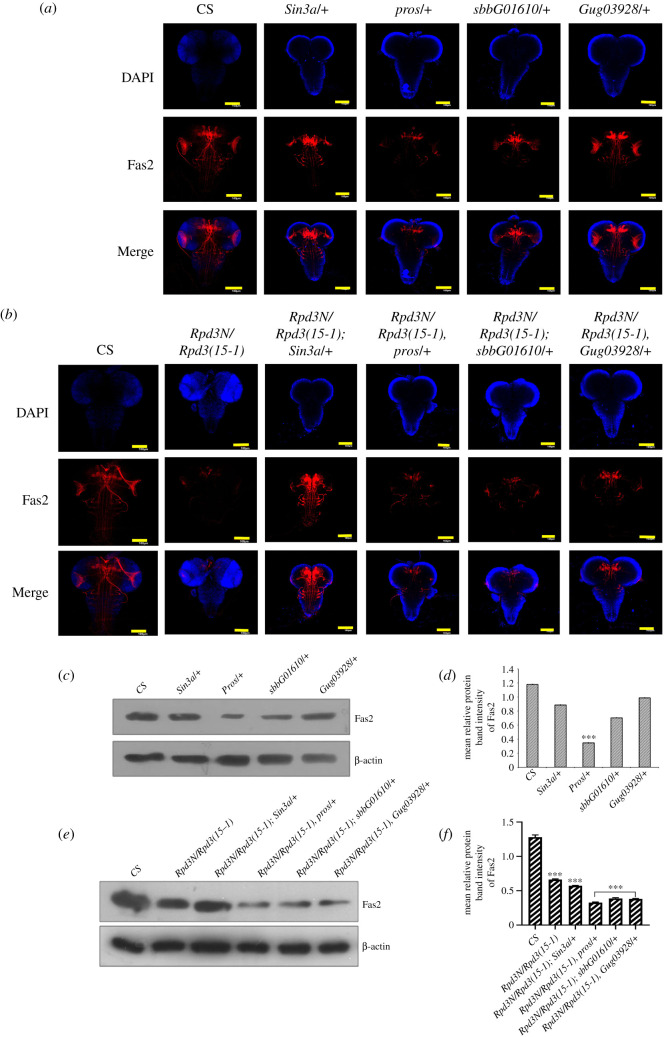
The variation in the expression of Fas2 in the mutant brains of the different interacting partners of Rpd3: (*a*,*b*) Crosses were set to obtain interacting genotypes between *Rpd3* and these interacting partners. The interacting genotype and individual mutant late third instar larval brains were immunostained with Fas2 antibody (red) to find out a variation in Fas2 expression. The images have been procured from *Z*-projections of the *XY* plane using a confocal microscope. Fas2 expression was further diminished in the heteroallelic mutants for *Rpd3* and also in the interacting genotypes for *Rpd3* and the interacting partners, Sin3A, prospero, scribbler and Atrophin. (*c*,*d*) The quantitative estimation of Fas2 in the late third instar larval brain (particularly in the mushroom bodies) were analysed in the interacting partners, Sin3a, prospero, Scribbler using western blot. The data were graphically represented as the mean relative intensity of protein band, after normalization of the internal control, β-actin, with error bars corresponding to s.e.m.; and ***p ≤ 0.001. The data are a representation of three independent experiments. (*e*,*f*) The western blot quantitative estimation of Fas2 in the late third instar larval brain (particularly in the mushroom bodies) was analysed in the different interacting genotypes and graphically represented as the mean relative intensity of protein band, after normalization of the internal control, β-actin, with error bars corresponding to s.e.m.; and ***p ≤ 0.001. Data are a representation of three independent experiments.

**Figure 8.**
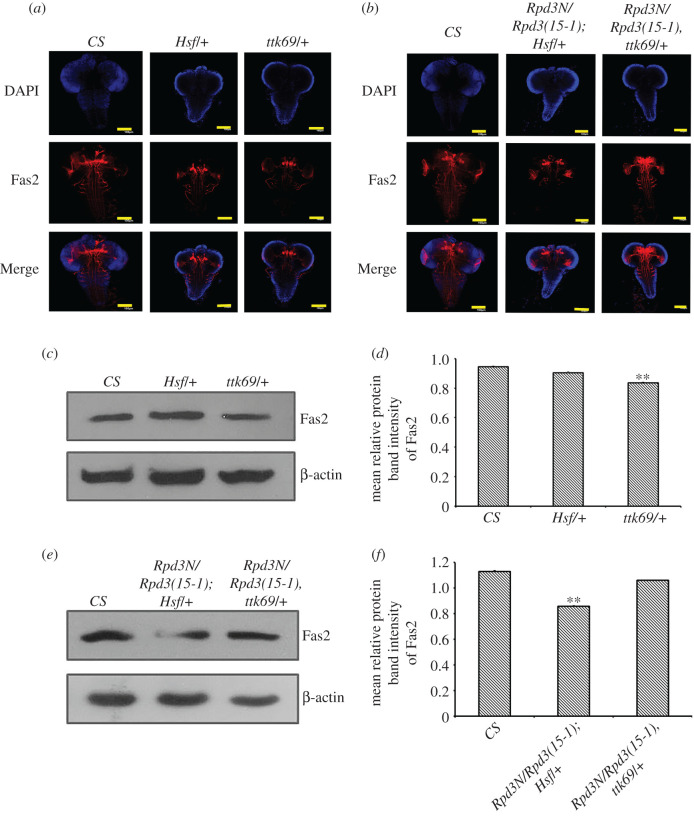
The variation in the expression of Fas2 in interacting genotypes for *Rpd3* along with *Hsf* and *ttk69*. (*a*,*b*) The late third instar interacting genotypes larval brains obtained through the crosses set between *Rpd3* and the interacting partner mutants for *Hsf* and *ttk69.* The larval brains were immunostained with Fas2 antibody (in red) and *Z*-projections of the brains in the *XY* plane were assessed. Scale bar, 100 µm. (*c*,*d*) The expression of Fas2 was quantified via the western blot analysis and represented graphically in the case of the individual interacting partners, *Hsf/+ and ttk69/+* compared to the wild-type (*CS*), post-normalization with internal control, β-actin (in the lower panel). The quantified protein intensity was graphically represented as the mean relative protein intensity, with error bars corresponding to s.e.m. with **p ≤ 0.01, wherein the data were obtained through three independent experiments. (*e*,*f*) The expression of Fas2 was quantified via the western blot analysis and represented graphically in the case of the interacting genotypes (*Rpd3N/Rpd3(15-1), ttk69/+*) and (*Rpd3N/Rpd3(15-1); Hsf/+)*, compared to the wild-type (*CS*) post-normalization with internal control, β-actin (in the lower panel). The quantified protein intensity was graphically represented as the mean relative protein intensity, with error bars corresponding to s.e.m. with **p ≤ 0.01, wherein the data were obtained through three independent experiments.

The Fas2 expression was further investigated in the mutants for the interacting partners of Rpd3 and also in the interacting genotypes for both Rpd3 and the respective interacting partners. The *Sin3a/+* mutants as well as the interacting genotypes (*Rpd3N/Rpd3(15-1); Sin3a/+*) showed negligible changes in Fas2 (figure 7*a*–*f*), while Fas2 reduced in the *pros/+* mutant, and the interacting genotypes (*Rpd3N/Rpd3(15-1), pros*) compared to the wild-type. In the *sbbG01610/+* mutant, Fas2 was almost close to that in the wild-type, but in the interacting genotypes (*Rpd3N/Rpd3(15-1); sbbG01610/+*), the expression was severely reduced (figure 7*a*–*f*). Fas2 showed no change in the *Gug03928/+* mutant but, in the interacting genotypes (*Rpd3N/Rpd3(15-1), Gug03928/+)*, there was a decrease in the expression (figure 7*a*–*f*). *Hsf*/+ alone did not conclusively reduce Fas2 but in (*Rpd3N/Rpd3(15-1); Hsf/+*), there was a very mild decrease in Fas2 signals compared to the wild-type. Similarly, *ttk69* when mutated individually or along with *Rpd3 (Rpd3N/Rpd3(15-1), ttk69/+)* did not reduce Fas2 significantly (figure 8*a–f*). Fas2 decreased in the interacting genotypes (*Rpd3N/Rpd3(15-1), Pc/+*) and (*Rpd3N/Rpd3(15-1); ph/+*) compared to the wild-type (electronic supplementary material, figure S2C,D). All these pieces of evidence led to the conclusion that *Rpd3* is more critical than the interacting partners for regulating the expression of Fas2 in larval brains.

### 3.7. The epidermal growth factor receptor negative regulators and Rpd3 control the expression of Tailless and Fas2

**Figure 9.**
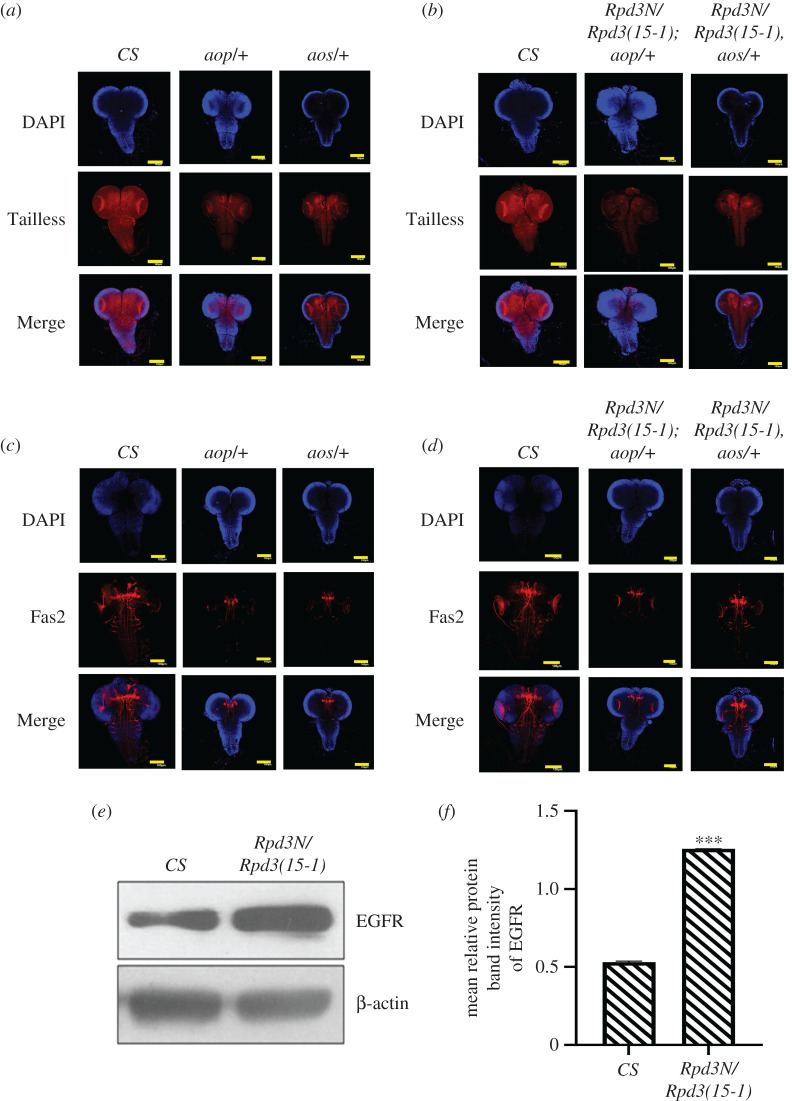
The variations in the expression of Tailless and Fas2 in the mutants for EGFR negative regulators, *argos* (*aos*) and *anterior open (aop),* and the histone deacetylase, *Rpd3*. (*a*,*b*) The expression of the EGFR pathway is negatively regulated by the repressors, Argos and Anterior open. Crosses were set between *Rpd3* and the negative regulators, *aos* and *aop*, and the late third instar larval progeny brains were immunostained with Tailless (in red) to find out the localization. The images are all *Z*-projections of confocal sections. The expression of Tailless was found to decrease in the *aos/+* and *yan*/+ and interacting genotype brains of (*Rpd3N/Rpd3(15-1), aos/+)* and *(Rpd3N/Rpd3(15-1); aop/+).* Scale bar, 100 µm. (*c*,*d*) The third instar progenies of the individual mutants for *aos* and *aop* and the interacting genotypes were immunostained with Fas2 (in red) and Z-projections of the stained brains were visualized to find out the localization. In the interacting genotypes for the interacting partners of EGFR- *aop* and *aos*, the expression of Fas2 showed reduction compared to the wild-type (here *CS*). Scale bar, 100 µm. (*e*,*f*) The quantitative western blot analysis was performed to compare the expression of dEGFR in the heteroallelic larval mutant brains for the histone deacetylase, *Rpd3*. The expression of dEGFR was quantitated against the expression of the internal control β-actin (in the lower panel) and graphically represented, with error bars corresponding to s.e.m. with ***p ≤ 0.001. The data are a representation of three independent experiments. There was an increase in the expression of dEGFR in the lysates obtained from the larval mutant brain of *Rpd3*, compared to the wild-type (*CS*).

The EGFR pathway is a critical regulator of the *Drosophila* optic lobe neurogenesis and drives the sequential progression of the proneural wave across the outer proliferation centres of the optic lobe [14,15]. EGFR expression is regulated by negative regulators, Aos and Aop*.* Aos regulates the Ras/MAPK pathway by binding to the epidermal growth factor while Aop inactivates the EGFR expression by negatively regulating transcription [62]. Tailless co-express with Fas2 in the optic lobes [5] and also antagonizes EGFR during the optic lobe formation [4] such that Fas2 decreases whenever there is a knockdown of *tailless* [5]. When the expression of Tailless was investigated in the *aop* and *aos* mutants individually and along with *Rpd3*, Tailless showed a mild decrease in the *aop*/+ mutants (figure 9*a*) and the (*Rpd3N/Rpd3(15-1); aop)* interacting genotypes (figure 9*b*). Tailless expression decreased in the *aos/+* and (*Rpd3N/Rpd3(15-1), aos/+*) mutants (figure 9*b*), compared to the wild-type (figure 9*a*). However, there was an almost equal decrease in Fas2 in the *aop/+* (figure 9*c*) as well as (*Rpd3N/Rpd3(15-1); aop/+*) mutants (figure 9*d*), while the *aos/+* mutant and the (*Rpd3N/Rpd3(15-1), aos/+*) interacting genotypes showed a greater reduction in Fas2 compared to the wild-type (figure 9*c,d*).

Because EGFR expression is known to be antagonized by Tailless [4], the western hybridization was performed to find out the quantitative changes in the EGFR expression in the *Rpd3* heteroallelic mutant larval brain*.* There was an increase in the EGFR expression in the *Rpd3* heteroallelic larval mutant brains compared to that of the wild-type (figure 9*e*,*f*). This concluded that in the *Rpd3* heteroallelic larval brains, there was an increase in EGFR expression, when the expression of Tailless, as well as Fas2, was reduced (figure 9*e,f*).

### 3.8. Rpd3 regulates the expression of different genes in neural development

**Figure 10.**
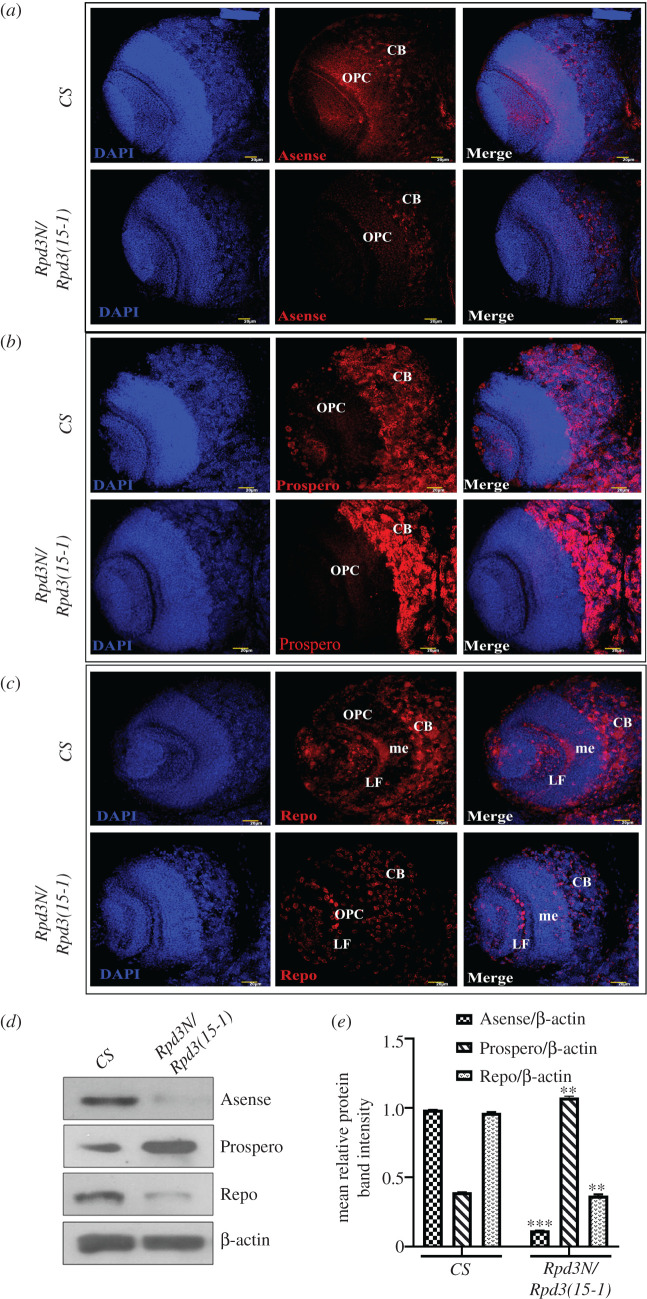
The expression of neural genes is under the regulation of Rpd3: *Asense, Prospero* and *Repo* constitute the genes involved in early neural development in *Drosophila*. The brain lobes of the wild-type (*CS*) and mutant larvae were examined under higher magnification (40x) and *Z*-projections of the same were procured to assess the localization of the proteins, in the outer proliferation centre (OPC) and central brain (CB). me, medulla; LF, lamina furrow. (*a*) Asense is the regulator of mitotic activity in the larval optic lobes. It serves as the marker of larval neuroblasts and is expressed in the newborn ganglion cells in the OPCs in the wild-type (CS). The heteroallelic mutant for *Rpd3* showed a decrease in the expression of Asense. It signified an upregulation of EGFR. Scale bar, 20 µm. (*b*) Prospero expresses in neural tissues undergoing differentiation and preventing mitotic activity. The heteroallelic mutant for *Rpd3* in the larval brain in *Z*-projections shows an increase in Prospero which signifies an upregulation of EGFR. Scale bar, 20 µm. (*c*) Repo provides the initial input for the neuroblast migration to form glial cells. The *Z*-projections from the *Rpd3* mutant showed a reduction in Repo expression. Scale bar, 20 µm. (*d*,*e*) The expression of Asense, Prospero and Repo in the mutant larval brains were quantitated using the western blot and represented graphically, after normalizing against β-actin and expressed as the mean relative protein density, from three independent experiments with three replicates each, with error bars corresponding to s.e.m. with ***p ≤ 0.001 and **p ≤ 0.01.

In *Drosophila*, the early neural development is controlled by genes like *asense (ase), prospero (pros)* and *reverse polarity (repo)*. The former observations made from this study have established that Tailless and Fas2 expressions are regulated by Rpd3. But no studies have been conducted yet to find out how these expressions are getting altered in the different mutants for *Rpd3*. The pan-neural and proneural gene, *asense*, is a mitotic regulator in the larval optic lobes whose loss-of-function increases proliferation and ectopic expression reduces proliferation [63]. The loss of function of *prospero* results in aberrant expression of cell cycle regulatory genes with premature cell cycle termination [64]. Repo is a glial-specific homeodomain protein [65] that expresses in the late glial cells [66,67]. The *tll*+ neuroblasts simultaneously express the gene, *glial cell missing* (gcm). The neuroblast progenies turn on the expression of Repo after *tll* is shut off. As a result of this, the progeny cells migrate towards the deeper neuronal layers and take up their final positions as glial cells around the medulla neuropils [68]. To investigate the changes in the expression of Asense, Prospero and Repo in the *Rpd3* mutant, larval brain immunostaining and western hybridization were performed*.* In the *Rpd3* heteroallelic mutant larval brains, the expressions of Asense and Repo decreased (figure 10*a,c*), while the expression of Prospero increased (figure 10*b*) compared to the wild-type. Results obtained from the western hybridization also substantiated that compared to wild-type, the expression of Asense and Repo were reduced (figure 10*d,e*), while the expression of Prospero was increased (figure 10*d,e*) which concluded that *Rpd3* regulates the expression of all the above neural development genes.

### 3.9. Fas2 expression gets altered in the eye disc for mutants of *Rpd3*, *aop* and *aos*

**Figure 11.**
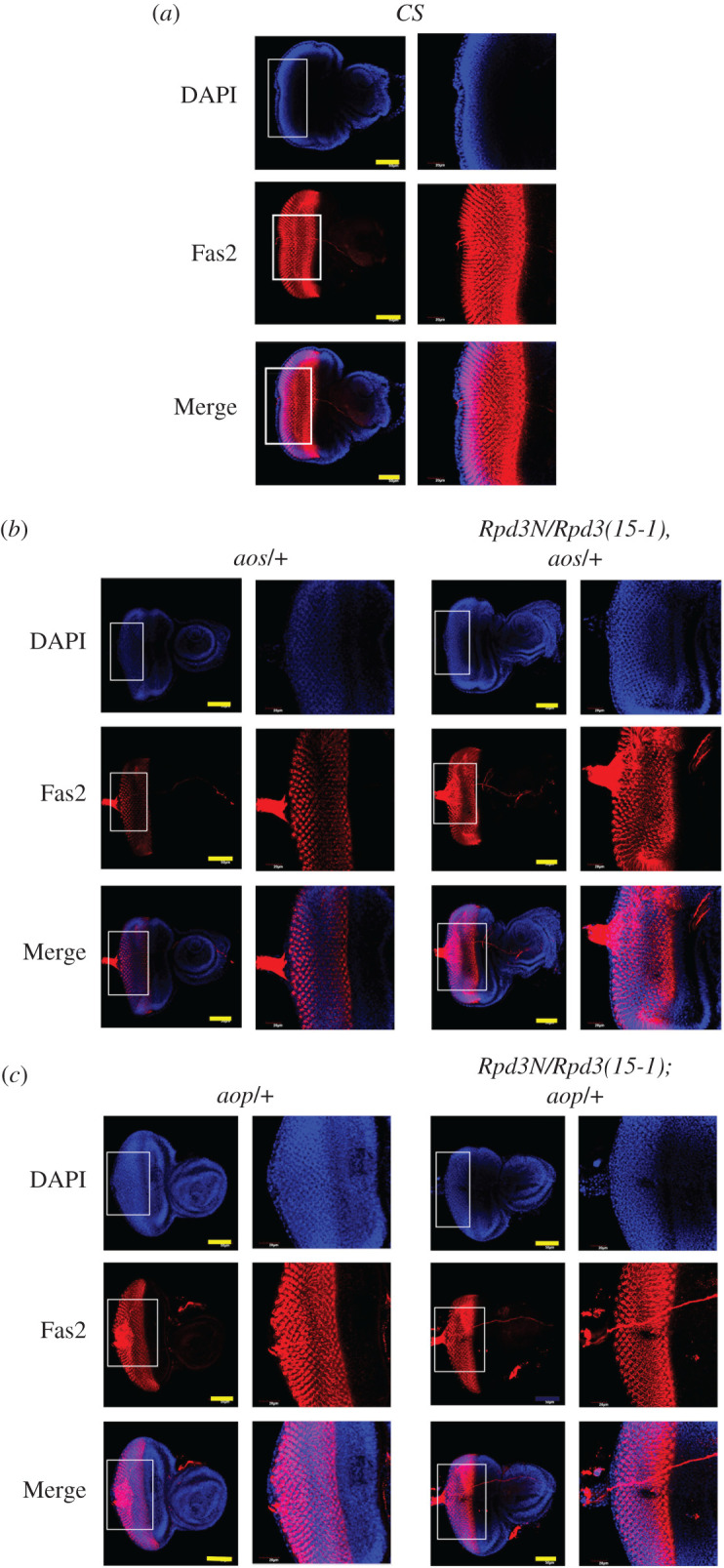
Fas2 expression undergoes alteration in the eye discs for mutants of *Rpd3*, *aop* and *aos*. (*a*) The third instar larval eye discs were dissected and immunostained with Fas2 (in red) to find out the localization pattern. The *Z*-projection depicts that Fas2 localized at the morphogenetic furrow of the eye disc, waning further to the posterior in the case of the wild-type (*CS*). (*b*) The *Z*-projection of eye discs of the interacting genotypes (*Rpd3N/Rpd3(15-1), aos/+*)*,* and the individual mutant *aos/+*, immunostained with Fas2 (in red) and observed under higher magnification. The expression of Fas2 alters in the eye disc showing a mild decrease in *aos/+* and (*Rpd3N/Rpd3(15-1), aos/+)* mutants. (*c*) The eye discs of the interacting genotypes (*Rpd3N/Rpd3(15-1);aop/+)* and the individual mutants *aop/+* were immunostained with Fas2 (in red) and observed under higher magnification captured in a *Z*-projection of the *XY* plane. Scale bars: 50 and 20 µm.

Fas2 exhibits a typical expression pattern in developing the eye in *Drosophila*. It never expresses in the undifferentiated cells anterior to the morphogenetic furrow but mainly localizes at the furrow, decreasing further to the posterior of the disc (figure 11*a*). Fas2 promotes EGFR signalling during the growth of the imaginal disc [69] that causes cell-specific expression of Fas2, suggesting a self-regulatory feedback loop between Fas2 and EGFR during eye disc development. Hence, the loss of *fas2* is reported to reduce the growth and proliferation of eye disc cells [69]. Aop repress EGFR and is degraded upon the Fas2 downregulation [20]. Aos negatively regulates the Ras/MAPK pathway that inhibits EGFR by preventing binding to Spitz [70]. When the expression of Fas2 was assessed in the eye disc for the mutants of *aos* and *aop*, as well as the *trans*-heterozygous mutants, Fas2 was found to decrease in the *aos/+* mutant and interacting genotypes (*Rpd3N/Rpd3(15-1), aos/+)* (figure 11*b*) as well as in the *aop*/+ and (*Rpd3N/Rpd3(15-1); aop/+)* mutants (figure 11*c*), compared to the wild-type. Thus, Fas2 regulation in the eye disc does not depend on the presence of Rpd3.

### 3.10. *Rpd3* regulates the embryonic size and ventral cuticular patterning via the epidermal growth factor receptor pathway

In zebrafish central nervous system, the histone deacetylase*, hdac1*, is critical for embryonic neurogenesis. A mutation in *hdac1* results in the reduction in the midbrain and anterior hindbrain size and produces a dorsally open spinal cord [71]. To investigate whether *Rpd3* assists in maintaining the normal size and volume of the *Drosophila* embryo, the width, length and volume of age-synchronized early developing embryos were analysed (electronic supplementary material, figure S3 and table S1). Both the width and length of the *Rpd3* heteroallelic embryos were found to vary by nearly 41–23% relative to the wild-type embryos. Lengths ranged from 0.57 mm in embryos from wild-type flies to 0.54 mm in *Rpd3N/ Rpd3(15-1)* (electronic supplementary material, table S1). This showed that histone deacetylase results in the maintenance of embryonic size (electronic supplementary material, figure S3) and that *Rpd3* regulate the size of the embryos.

The *Drosophila* larvae patterning was studied by examining the ventral epidermis. The ventral epidermal cells secrete short, stout hair-like projections called denticles, under the influence of the EGFR pathway. These exist in the repetitive belts of rows at the anterior half of each parasegment, along the anteroposterior axis. The antagonism between the EGFR and *Wingless* signalling decides the basic cell fate of the alternating belts of denticle and smooth cuticle [72,73]. To find out if a mutation in *Rpd3* altered the ventral cuticular patterning, the *Rpd3* heteroallelic third instar mutant larvae were examined at 27× and 150× magnifications, respectively. The first four rows of denticles were found to be prominently developed in the *Rpd3* heteroallelic mutant larvae, compared to the wild-type (electronic supplementary material, figure S4). The result signified that *Rpd3* mutation produces moderate changes in the larval denticle patterning.

## 4. Discussion

The present study deals with the regulation of Tailless by Rpd3 during early embryonic development and the effect of *Rpd3* and the interacting partners on Tailless expression in the *Drosophila* larval brain.

The histone deacetylase, dHDAC4, is a known regulator of the Gap and Pair-Rule genes in *Drosophila* [74] that made us investigate for a possible role of the *Drosophila* histone deacetylase, Rpd3 in the gap genes regulation*.* Rpd3 has reported roles of repression with the interacting partners (co-repressors) Sin3a [75], prospero [12] and Atrophin [76]. The initial studies involving immunostaining and western hybridization investigated the changes in the gap gene expression in the *Rpd3* heterollelic embryos and showed a complete loss of Tailless, signifying that Rpd3 has an important role to execute in the maintenance of normal expression of Tailless. However, *Rpd3* did not affect the expression of Kruppel and Giant probably because these genes receive inputs from both the maternal genes (mainly *bicoid*) and primary gap genes to sustain their normal expression. Hunchback is present both maternally and zygotically (regulated by inputs from Bicoid). Because Rpd3 did not affect the Bicoid expression, there was no change in the expression of Hunchback, Kruppel and Giant in the *Rpd3* heteroallelic mutants (figure 1).

The ChIP assay was performed to ascertain the enrichment of the common epigenetic markers (H3K9me3, H3K27me3, RNA Pol II, H3K9ac and H3K4ac) in the promoter region of *tailless*. H3K27 is highly enriched and acetylated at high levels in the early embryos and there is an increased enrichment of methylated H3K27 only after 4 h after egg laying [52]. H3K27 undergoes acetylation by CREB-binding protein (CBP) and deacetylation by Rpd3 [77]. There was a decreased enrichment of H3K27me3 in the *Rpd3* mutant embryos compared to the wild-type (figure 2*b*). Because deacetylation, which involves the removal of an acetyl group from the histone tail of H3K27 requires Rpd3, the loss-of-function of *Rpd3* may have resulted in a reduced enrichment of H3K27me3. A severely decreased enrichment of euchromatic markers, H3K9ac and H3K4ac along with a very significant low enrichment of RNA polymerase II, was observed at the promoter region of *tailless* (figure 2*b*). The occupancy of RNA Pol II at the promoter of a gene primes genes for getting activated during early development [78]. H3K9ac prevents the RNA Pol II from escaping promoter-proximal pausing on chromatin, and H3K4ac mainly localize and enrich in the promoters of the active genes [47,48]. The high degree of reduction in the RNA Pol II in the *tailless* promoter (figure 2*b*) accounts for the decrease in the expression that was evident from the immunostaining as well as the western blot results (figure 1). Thus, the above study established the importance of Rpd3 in the Tailless regulation in embryos.

Besides forming terminal domains during *Drosophila* segmentation [79], Tailless also regulates early brain development. It serves as a dedicated transcriptional repressor in early *Drosophila* embryos [80]. Tailless reportedly interacts with Atonal (ato), Sine oculis (so), Eyeless (ey) and Eyes absent (eya), and the EGFR signalling pathway to establish the *Drosophila* embryonic visual system [4]. Therefore, Tailless was chosen to serve as readout for analysing the mechanism of regulation employed by Rpd3 during larval brain development. The loss of Tailless expression in the *Rpd3* heteroallelic larvae (figure 3*a*,*c*,*d*) showed that it has a critical role in larval brain development. The significant decrease in the expression of *Rpd3* and *tailless* mRNAs in the *Rpd3* heteroallelic third instar larval brains compared to the wild-type (*CS*) demonstrated that *Rpd3* heteroallelic mutation decreases the expression of *tailless* mRNA (figure 4*a*,*b*).

The interacting partners of Rpd3 include Sin3a, prospero, Atrophin, scribbler and polycomb proteins. The expression of Tailless decreased in the larval brain of *Sin3a/+* and (*Rpd3N/Rpd3(15-1), Sin3a/+)* mutants (figure 5*a*–*f*). Sin3a is a co-repressor of Rpd3; however, upon the loss-of-function mutation of *Sin3a*, there was a decrease in the expression of Tailless. This raises the possibility that the other interacting partners are more effective in maintaining the repressed state of *tailless*, even in the absence of *Sin3a*. Atrophin interacts with Rpd3 via H3 acetylation on the N terminus forming a trimeric complex that reduces *decapentaplegic* transcription [76]. Atrophin co-represses *Even-skipped* [11,81] and genetically interacts with Tailless, in addition to the physical interaction the ligand-binding domain of Tailless. A higher decrease in Tailless in the interacting genotypes (*Rpd3N/Rpd3(15-1), Gug03928/+*) signified that Atrophin (figure 5*b*,*e*,*f*) prevented Tailless expression by hindering DNA-binding and transcription. Scribbler, another transcriptional repressor of Tailless, was found to repress Tailless expression in the interacting genotypes (*Rpd3N/Rpd3(15-1); sbbG01610/+*) (figure 5*a*–*f*) which suggests that Rpd3 plays a crucial role in Tailless expression.

Ttk69 represses Tailless [61] by binding to the 3' flanking region of tor-RE. Hsf activates Tll by getting phosphorylated at S378, transforming from a repressor to an activator and, in this process, relieves the repression of Tailless [82]*.* The larval mutants where both *Rpd3* and *Hsf* were mutated (*Rpd3N/Rpd3(15-1); Hsf/+)* showed no changes in Tll expression (figure 6*a–f*) but in (*Rpd3N/Rpd3(15-1), ttk69/+)*, there was a decrease in Tll (figure 6*a–f*). The decreased expression of Tll in (*Rpd3N/Rpd3(15-1), ttk69/+*) mutants can be attributed to the failure of the GAF/Hsf/Ttk69 complex formation, owing to the absence of Rpd3. The failure to form the complex between Ttk69, Sin3a and pits (protein interacting with Ttk69 and Sin3a) protein simultaneously results in the loss of Tailless. This highlighted the necessity of both Rpd3 and Ttk69 to be present simultaneously for the normal Tailless expression in the *Drosophila* larval brain. The finding that Tailless along with Rpd3 is essential for normal brain development was thus confirmed and further reinforced by ruling out the roles of the interacting partners of Rpd3 and Tailless.

In the *Rpd3* heteroallelic mutant, the expression of Fas2 has reduced in comparison to its wild-type counterpart (figure 3*b–d*). In all the interacting genotypes for the partners of Rpd3 or Tailless, Fas2 expression was found to be completely dependent on the presence of Rpd3. There were evident similarities in the expression of Fas2 as well as Tailless in the respective interacting genotype of *Rpd3* and the interacting partners (figures 7*a–f* and 8*a–f*). The known fact that both Fas2 and Tailless co-localize in the larval brain could justify such similarities.

Aos and Aop are negative regulators of EGFR which gets activated when EGFR production needs to be shut down. Whenever these negative regulators are mutated, EGFR levels increase. There was a significant decrease in Tailless in the larval brains of the *aop/+* (figure 9*a*) and (*Rpd3N/Rpd3(15-1); aop/+)* mutants (figure 9*b*) as well as in the larval brains of the *aos/+* (figure 9*a*) and the (*Rpd3N/Rpd3(15-1), aos/+*) mutants (figure 9*b*). Fas2 decreased almost equally in the larval brains for all the mutants of Aop and Aos compared to wild-type (figure 9*c*,*d*). Previous reports suggest that during optic lobe formation, EGFR signalling is antagonized by Tailless [4]. Therefore, the decrease in Tailless expression in the mutants for *Rpd3* as well as *aop* and *aos* may contribute to the EGFR upregulation. Fas2 interacts with EGFR genetically and inhibits EGFR for normal eye development. The loss-of-function of *fas2* degrades Aop to cause EGFR hyperactivation in the eye, notum and wing [20]. In the mutants for *aos* and *aop,* Fas2 decrease may contribute to the increase in EGFR, correlating with the Tailless expression in these mutants. Therefore, to assess this, we checked for EGFR protein levels in the *Rpd3* mutant larval brains. The western blot showed an upregulation of the dEGFR expression in the *Rpd3* heteroallelic larval mutant compared to wild-type (figure 9*e*,*f*) which substantiated the above results and concluded that in the *Rpd3* heteroallelic larval mutant brains, a robust decrease in the expression of Tailless and Fas2 increased the expression of dEGFR.

EGFR is crucial for neurogenesis in *Drosophila* and generates the proneural wave as a downstream result of EGFR activation [83]. The proneural gene then initiates the expression of the pan-neural genes—*asense* [84,85] and *Prospero* [86,87]*. Asense* is a pan-neural homeobox gene that is activated by the proneural genes when EGFR is downregulated. It upregulates the neuroblast genes and represses the neuronal differentiation genes [88]. The expression of Asense was found to be downregulated in the *Rpd3* heteroallelic mutant larval brain which signified that EGFR was upregulated in this mutant (figure 10*a*,*d*,*e*). *Prospero* is a neuronal precursor gene that shares an antagonistic role to *Asense*. It represses the neuroblast genes and upregulates the neuronal differentiation genes [89]. Prospero expresses as a result of EGFR activation by the binding of the EGFR ligand to the precursor cells [90,91]. Prospero upregulation in the *Rpd3* mutant also indicates a higher EGFR expression (figure 10*b*,*d*,*e*). *Repo* is a glial cell differentiation gene that initiates the neuroblast migration to form the glial cells [66]. EGFR is critical for the survival of the glial cells via the ligands—Spitz and Vein [92]. EGFR signalling promotes neural fate by repressing the genes, *repo* and *gcm*, responsible for promoting a glial cell identity. In the *Drosophila* central nervous system, the loss of EGFR causes glial cells to undergo apoptosis [93,94]. The expression of Repo in the *Rpd3* mutant was reduced compared to the wild-type which indicates the upregulation of EGFR (figure 10*c–e*). Thus, the alteration in the expression of all the neural genes serves as readouts to justify that there has been an upregulation of EGFR signalling in the *Rpd3* mutant larval brain.

Fas2 expression negligibly decreased in the *aos/+* and (*Rpd3N/Rpd3(15-1), aos/+)* mutants larval eye discs (figure 11*b*) as well as in the *aop/+* and (*Rpd3N/Rpd3(15-1); aop/+)* mutants compared to the wild-type (figure 11*c*). All these results concluded that the regulation of Fas2 expression in the eye disc is not dependent on the presence of Rpd3.

In *Drosophila*, the pro-apoptotic gene head involution defective (*hid*) mediates apoptosis in the loss-of-function mutants for *bicoid* [95], *tailless* and *Kruppel* [95]. During mid-embryogenesis, EGFR ligands are produced in response to the segmentation cascade that inhibits hid in the epidermis, creating survival signals. After stage 11, EGFR activates hid [96] to prune off the excess cells to restore the correct dimensions of the embryo [19]. EGFR loss-of-function stimulates cells in the anterior placode to undergo apoptosis, reducing the placode size [97]. The volume of the heteroallelic *Rpd3* mutant embryos decreased in comparison to the wild-type probably because EGFR was downregulated (electronic supplementary material, figure S3 and table S1). However, the expression of EGFR here is counterintuitive to the increase in EGFR in the *Rpd3* mutant larval brain.

The denticles in rows 1–4 at the ventral epidermis of a larva are specified by the EGFR pathway, with simultaneous inhibition of the *Wingless* signalling pathway [72,73] which cumulatively regulate the transcription of *shavenbaby*. A high level of EGFR directly activates shavenbaby producing denticles, while *wingless* represses the expression of shavenbaby, producing naked cuticles [73]. When the ventral epidermis of the third instar mutant larvae were examined, there were a well-defined first four rows of denticle belts in the mutant larvae. This signified that EGFR which is responsible for initiating denticle growth was upregulated possibly leading to the activation of *shavenbaby*. This result is, therefore, in accordance with the previous findings which also highlighted that there is an upregulation of EGFR in the heteroallelic *Rpd3* mutants (electronic supplementary material, figure S4).

## 5. Conclusion

**Figure 12.**
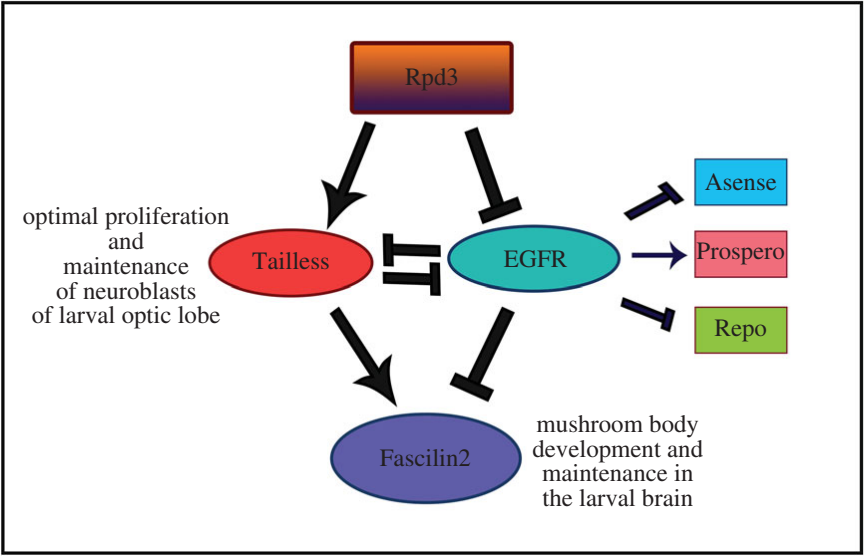
Summary of the Rpd3–Tailless–dEGFR axis regulating the larval brain development in *Drosophila*. In a nutshell, Rpd3 acts as an activator of Tailless and Fascilin2, which, respectively, control the optimal proliferation of neuroblasts of the optic lobe and development of the mushroom body progenitor cells in the *Drosophila* larval brain. dEGFR (written as EGFR) is inhibited by Rpd3, while dEGFR itself inhibits the expression of Fascilin2. Repo and Asense undergo inhibition by dEGFR activation while Prospero is activated in the presence of dEGFR.

This novel work, therefore, provides unique insights about the effect of mutations of the histone deacetylase, *Rpd3* on Tailless expression in the early stages of embryo and larval brain development. The gap gene, *tailless*, belongs to the family of the orphan nuclear receptor, with established significant roles in the development of the terminal region and brain. However, to date, to our knowledge there have been no earlier reports on the regulation of Tailless by the histone deacetylase, Rpd3, in the larval brain. The analysis of Tailless and Fas2 expression in the larval brain depicted that Rpd3 needs to be present with the interacting partners for the sustenance of the normal expression of Tailless and Fas2. It proves that the expression of Tailless in the larval brain and embryo is regulated by the EGFR signalling pathway. The EGFR pathway is already known to be important for the normal development of the larval brain and eye. This study provides new insights into the Tailless regulation, highlighting the role of Rpd3 in regulating EGFR expression during larval brain development. The increased expression of EGFR in the brain, particularly in the larval mutant of *Rpd3* showed that Rpd3 plays a critical role in downregulating the EGFR expression and maintaining the normal expression of Tailless. Thus, it provides novel insight into the Tailless regulation in the early embryonic and larval development via the EGFR pathway (figure 12).

## Data accessibility

This article has no additional data.
