# Correction to ‘Histone deacetylase (Rpd3) regulates *Drosophila* early brain development via regulation of Tailless’

**DOI:** 10.1098/rsob.200323

**Published:** 2020-10-21

**Authors:** Paromita Das, Manika Pal Bhadra

*Open Biol.* **10**, 200029 (Published Online 2 September 2020) (doi:10.1098/rsob.200029)

This correction refers to an error in the affiliation of author Paromita Das. The affiliation should read as follows - Academy of Scientific and Innovative Research (AcSIR), Ghaziabad- 201002, India.

